# Functional connectivity changes associated with fMRI neurofeedback of right inferior frontal cortex in adolescents with ADHD

**DOI:** 10.1016/j.neuroimage.2018.11.055

**Published:** 2019-03

**Authors:** K. Rubia, M. Criaud, M. Wulff, A. Alegria, H. Brinson, G. Barker, D. Stahl, V. Giampietro

**Affiliations:** a Department of Child and Adolescent Psychiatry, Institute of Psychiatry, Psychology and Neuroscience, King's College London, UK; b Department of Neuroimaging, Institute of Psychiatry, Psychology & Neuroscience, King's College London, UK; c Department of Biostatistics & Health Informatics, King's College London, UK

**Keywords:** ADHD, Functional connectivity, fMRI-neurofeedback, Default mode network (DMN), Cognitive control network, Inferior frontal cortex

## Abstract

Attention Deficit Hyperactivity Disorder (ADHD) is associated with poor self-control, underpinned by inferior fronto-striatal deficits. We showed previously that 18 ADHD adolescents over 11 runs of 8.5 min of real-time functional magnetic resonance neurofeedback of the right inferior frontal cortex (rIFC) progressively increased activation in 2 regions of the rIFC which was associated with clinical symptom improvement. In this study, we used functional connectivity analyses to investigate whether fMRI-Neurofeedback of rIFC resulted in dynamic functional connectivity changes in underlying ***neural networks.***

Whole-brain seed-based functional connectivity analyses were conducted using the two clusters showing progressively increased activation in rIFC as seed regions to test for changes in functional connectivity before and after 11 fMRI-Neurofeedback runs. Furthermore, we tested whether the resulting functional connectivity changes were associated with clinical symptom improvements and whether they were specific to fMRI-Neurofeedback of rIFC when compared to a control group who had to self-regulate another region.

rIFC showed increased positive functional connectivity after relative to before fMRI-Neurofeedback with dorsal caudate and anterior cingulate and increased negative functional connectivity with regions of the default mode network (DMN) such as posterior cingulate and precuneus. Furthermore, the functional connectivity changes were correlated with clinical improvements and the functional connectivity and correlation findings were specific to the rIFC-Neurofeedback group.

The findings show for the first time that fMRI-Neurofeedback of a typically dysfunctional frontal region in ADHD adolescents leads to strengthening within fronto-cingulo-striatal networks and to weakening of functional connectivity with posterior DMN regions and that this may be underlying clinical improvement.

## Graphical abstract

**Figure:**
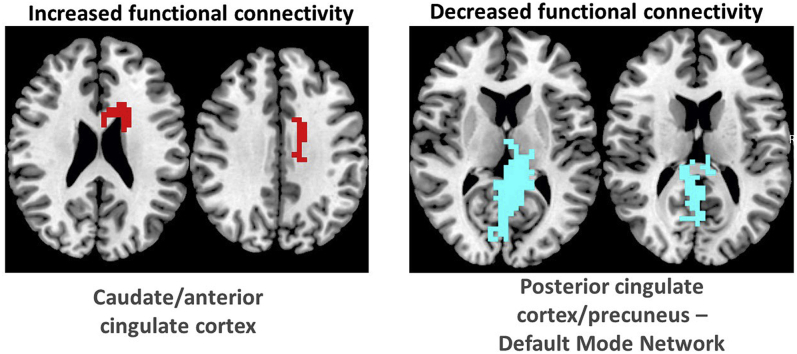

## 1 Introduction

Attention Deficit Hyperactivity Disorder (ADHD) is a highly prevalent (around 7% prevalence worldwide) and male-predominant (4:1) childhood disorder of age-inappropriate problems with inattention, impulsiveness, and hyperactivity, that persists into adulthood in most cases (Thomas et al., 2015). Psychostimulant medication, the gold-standard treatment for ADHD, is associated with significant symptom improvements in about 70% of patients (Stevens et al., 2013). While superior to behavioural treatments after 14 months, longer-term efficacy of medication has not been demonstrated (Cunill et al., 2016; Molina et al., 2009) which may be related to evidence for dopaminergic brain adaptation to psychostimulant medication (Fusar-Poli et al., 2012; Wang et al., 2013). Other limitations include adverse effects, restricted use for certain comorbid conditions, potential for abuse and diversion, unknown longer-term brain effects, and limited compliance in adolescence. Therefore, non-pharmacological treatments such as diets, behavioural or cognitive training are preferred, but have shown limited efficacy (Sonuga-Barke et al., 2013).

Brain-based therapies such as real time fMRI-neurofeedback can target the key underlying neurofunctional deficits in ADHD and are therefore promising (Rubia, 2018a). fMRI-Neurofeedback is based on operant conditioning and teaches participants to self-regulate blood-oxygen level-dependent (BOLD) response in specific brain regions based on real-time feedback of their brain activation which can be gamified in an attractive and engaging way for children.

The advantages of fMRI-Neurofeedback are no known side effects and potential longer-term neuroplastic effects. Electrophysiological neurofeedback (EEG-Neurofeedback) in ADHD, which targets abnormal EEG biomarkers, has in fact shown longer-term effects of up to 2 years (Gevensleben et al., 2010; Van Doren et al., 2018; Strehl et al., 2006). However, recent meta-analyses and reviews of randomized controlled trials of EEG-Neurofeedback of “probably” blinded raters show only trend-level improvements (Holtmann et al., 2014; Thibault and Raz, 2017). Neurofeedback using fMRI has several advantages over EEG-Neurofeedback. Due to its superior spatial resolution it can target key neurofunctional biomarkers established over the last 2 decades of fMRI research, such as the inferior frontal cortex or the basal ganglia, which cannot directly be reached with EEG-Neurofeedback (Rubia, 2018a). Although more costly per session, self-regulation is typically achieved much faster than with EEG-Neurofeedback, where in ADHD typically 30–40 hourly runs of 50 min are used (Arns et al., 2009). Healthy adults can self-regulate specific brain activity in 4 runs of 8 min within one fMRI session (Lawrence et al., 2014; Rota et al., 2009) and in our fMRI-Neurofeedback study we showed that ADHD adolescents can learn to enhance specific brain activity on average in 8 runs of 8.5 min (Alegria et al., 2017), which is a substantially faster self-regulation than that achieved with EEG-Neurofeedback. Last, a key advantage of fMRI-Neurofeedback over EEG-Neurofeedback is that it allows the investigation of the effects of self-regulation of a specific region on entire brain networks by using functional connectivity analyses (Emmert et al., 2016; Thibault et al., 2015, 2016, 2017).

Despite very promising effects of fMRI-Neurofeedback in other disorders (Thibault et al., 2017), only two studies have tested fMRI-Neurofeedback in ADHD. A pilot study in 7 adults with ADHD tested fMRI-Neurofeedback of the dorsal anterior cingulate cortex over 4 hourly MRI sessions while performing a mental calculation task expected to increase dorsal anterior cingulate cortex activation, compared to 6 ADHD adults who did not receive fMRI-Neurofeedback. The study found that although both groups showed similar dorsal anterior cingulate cortex activation increases during training and transfer runs, ADHD symptoms were not improved in either group. However, the active, but not the control group, showed performance improvements in sustained attention and working memory (Zilverstand et al., 2017). The second study, from our lab, tested fMRI-Neurofeedback in 31 ADHD adolescents in a randomized controlled trial where the active target group (N = 18) had to learn to upregulate the rIFC, while the control group (N = 13) had to upregulate a control region, the left parahippocampal gyrus (lPHG) in 11 runs of 8.5 min of fMRI-Neurofeedback over 4 hourly scans over 2 weeks using a rocket movie as feedback (Alegria et al., 2017). The fMRI data showed significantly enhanced linear activation increase in two regions of the rIFC across all 11 sessions in the rIFC-Neurofeedback relative to the control group and enhanced linear activation increase in 3 regions of the lPHG in the control relative to the rIFC-Neurofeedback group. Only the rIFC-Neurofeedback group, however, showed significant transfer effects (increased activation in the target region when no feedback was provided which is considered a proxy for transfer of learned self-regulation to daily-life), which furthermore were significantly associated with a reduction of clinical ADHD symptoms. Although both groups improved significantly in the clinical ADHD severity measure, only in the rIFC-Neurofeedback group they were correlated with the brain changes, thus demonstrating brain-behaviour associations, arguing against a mere placebo effect. Effect sizes were medium (0.6) at post-assessment for both groups, but large in the rIFC-Neurofeedback group (almost 1) at 11 months follow-up with a 26% reduction in ADHD symptoms relative to only trend-level significant medium effect size changes of 16% symptom reduction in the control group, suggesting longer-term and potentially delayed consolidation effects of fMRI-Neurofeedback of rIFC in the active group (Alegria et al., 2017). In addition, only the rIFC-Neurofeedback group showed trend-level improved sustained attention performance and increased inhibitory brain activation during a stop task relative to the control group. Although we cannot rule out placebo effects, the rIFC-Neurofeedback treatment had several advantages over the control treatment, such as stronger brain-behaviour correlations, exclusive transfer effects, exclusive cognitive improvements, stronger longer-term effects, and exclusive brain activation benefits in the fMRI stop task relative to the control group.

As mentioned above, a main advantage of fMRI-Neurofeedback is the possibility to investigate the effects of Neurofeedback of a specific brain region on the activation of the entire brain and on dynamic functional neural networks.

In the secondary analysis we report here, we were particularly interested in exploring the mechanism of action of self-regulation of rIFC with fMRI-Neurofeedback in ADHD on underlying dynamic ***neural networks.*** For this purpose, we conducted seed-based whole brain functional connectivity analyses on the data from the rIFC-Neurofeedback group (who had to enhance activity in rIFC). We aimed to test whether rIFC self-regulation is associated with increased or decreased functional connectivity with other regions of the brain. Several fMRI-Neurofeedback studies have shown that the training of self-regulation of specific frontal regions leads to the co-activation and increased functional connectivity with other interconnected regions (Hui et al., 2014; Papoutsi et al., 2018; Rota et al., 2009; Sarkheil et al., 2015; Sepulveda et al., 2016; Zhang et al., 2015a); for review see (Thibault et al., 2017). Given that the rIFC is a cognitive control hub region mediating functions compromised in ADHD, such as cognitive control and attention (Hugdahl et al., 2015; Kim, 2014; Radua et al., 2014) and is part of the cognitive control network (Arnsten and Rubia, 2012; Beckmann et al., 2005; Damoiseaux and Greicius, 2009; He et al., 2009; Moussa et al., 2012; Sripada et al., 2014b), we hypothesised that self-upregulation of the rIFC in ADHD patients would be associated with enhanced functional connectivity of the rIFC with other areas that form part of the cognitive control network, such as the dorsal anterior cingulate cortex, the pre-supplementary motor area and the basal ganglia (Arnsten and Rubia, 2012; Guo et al., 2018; He et al., 2009; Niendam et al., 2012).

Furthermore, based on consistent evidence that the activation of task-positive regions, in particular cognitive control hub regions such as the rIFC, is associated with the deactivation and hence anti-correlated with activation in DMN regions (Broyd et al., 2009; Fox et al., 2005; Raichle, 2015; Raichle et al., 2001; Sripada et al., 2014a), we hypothesised that rIFC upregulation through fMRI-Neurofeedback would be associated with increased deactivation and hence decreased functional connectivity with regions of the DMN such as ventromedial frontal cortex, posterior cingulate, precuneus and inferior temporo-parietal regions (Broyd et al., 2009; Fox et al., 2005; Raichle, 2015; Raichle et al., 2001; Sripada et al., 2014a). Meta-analyses of fMRI studies of cognitive control have shown that ADHD is associated most consistently with poor activation in cognitive control regions such as the rIFC, basal ganglia, anterior cingulate cortex and the supplementary motor area (Hart et al., 2013; Norman et al., 2016) as well as with poor deactivation of DMN regions such as the ventral anterior cingulate cortex and posterior cingulate and precuneus during cognitive tasks (Rubia, 2018a,b; Christakou et al., 2013; Fassbender et al., 2009; Hart et al., 2012, 2013; Salavert et al., 2015), which were furthermore significantly anti-correlated with poor fronto-striatal activation (Christakou et al., 2013). A connectomic study of a large multi-site resting state dataset (ADHD200) in 7–21 year olds, furthermore found an age by ADHD severity interaction in 133 ADHD patients relative to 288 healthy controls, suggesting that ADHD patients have a maturational lag in the connectivity ***within*** the ventral attention network, the fronto-parietal cognitive control network, and the DMN, as well as in the anti-correlation ***between*** these task-positive networks and the DMN (Sripada et al., 2014a). Functional connectivity deficits furthermore have been found in several other neural networks in relation to ADHD such as motor, saliency, cerebellar and reward-based networks (Castellanos and Aoki, 2016; O'Halloran et al., 2018; Rosenberg et al., 2016). It has been argued, therefore, that ADHD patients have less control over their interoceptive attention orientation and mind-wandering, which intrudes more into their already weak exteroceptive attention and cognitive control processes, likely causing enhanced inattention and impulsiveness. This immature pattern of poor activation of task-relevant networks and of decreased deactivation of the DMN is furthermore likely underlying the poor performance in ADHD patients on attention-demanding higher-level cognitive control tasks (Rubia, 2018a; b; Rubia et al., 2014a).

As a consequence, we hypothesised that the training of the progressive upregulation of the right IFC in ADHD adolescents with fMRI-Neurofeedback would result in both positive and negative functional connectivity changes with task-positive and task-negative networks, respectively. Specifically, we hypothesised that 1) fMRI-Neurofeedback of rIFC would lead to enhanced functional connectivity after relative to before Neurofeedback training between rIFC and other areas of the cognitive control network that are typically underactivated in ADHD such as the dorsal caudate, the dorsal anterior cingulate cortex and the pre-supplementary motor area (Hart et al., 2013; Norman et al., 2016; Niendam et al., 2012); and 2) that rIFC upregulation would be associated with decreased functional connectivity between the rIFC and areas of the DMN such as posterior cingulate, precuneus, inferior temporo-parietal regions and ventromedial prefrontal cortex.

Furthermore, we hypothesised that the increased positive functional connectivity post-treatment between the rIFC and striatal and anterior cingulate cortex/supplementary motor area regions, and the reduced functional connectivity between the rIFC and areas of the DMN would be associated with a reduction in ADHD symptoms and be specific to the fMRI-Neurofeedback group that had to enhance the rIFC relative to the control group that had to self-upregulate the lPHG.

## 2 Material and methods

The experimental design has been previously described in (Alegria et al., 2017). The original study was a randomized controlled trial testing the efficacy of fMRI-Neurofeedback of rIFC in 18 ADHD adolescents compared to a group of 13 ADHD adolescents who had to enhance another control region, the left parahippocampal gyrus.

In the current study, we were particularly interested in the effects of rIFC self-regulation training in ADHD on functional connectivity with other brain networks. For this purpose, the rIFC-Neurofeedback group is the main focus of the study. However, to assess specificity of the functional connectivity findings in the rIFC-Neurofeedback group, functional connectivity changes in the control group are also reported in the text and supplement.

### 2.1 Participants

Eighteen right-handed (Oldfield, 1971) boys (12–17 years old; mean (SD) = 14 (2)) were recruited from South London clinics. They all met the clinical DSM-5 criteria for the diagnosis of ADHD, combined hyperactive/impulsive and inattentive (N = 16) or inattentive (N = 2) subtypes, as assessed by an experienced child psychiatrist and confirmed with the Schedule of Affective Disorders and Schizophrenia for School-Age Children-Present and Lifetime version (K-SADS-PL) (Kaufman et al., 1996). They also scored above clinical ADHD threshold on the Conners' Parent Rating Scale (CPRS-R), a parent rated index of ADHD severity (Conners et al., 1998). The Social Communication Questionnaire (Rutter et al., 2003) screened for autism spectrum disorders. Two boys met/exceeded the cut-off score of 15, but a possible autism spectrum condition was ruled out by clinical interview. General functioning and symptom severity were assessed with the Children's Global Assessment Scale (Shaffer et al., 1983).

13 control ADHD patients (mean age (SD) = 14 (2)) were recruited and assessed in the same way, meeting DSM-5 criteria for the diagnosis of ADHD, combined hyperactive/impulsive and inattentive (N = 11) or inattentive (N = 2) subtypes.

Exclusion criteria for all were IQ < 80 (Wechsler, 1999) alcohol or substance abuse, neurological or comorbid psychiatric disorders, except for disruptive behaviour disorder, and MRI contraindications. Fifteen boys of the rIFC-Neurofeedback group and 9 control boys of the lPHG-Neurofeedback group received stable psychostimulant administration throughout the fMRI-Neurofeedback period. Baseline testing started at least seven days after titration (rIFC-Neurofeedback group: methylphenidate: N = 13, dexamphetamine: N = 2; control group: methylphenidate N = 9). Three patients in the rIFC-Neurofeedback and control groups each ceased taking medication for at least seven days before baseline testing and one control boy was stimulant-naïve. The study was approved by the local ethics committee and conducted in accordance with the Declaration of Helsinki (Research Ethics Committee reference number: 12/LO/0708). Written informed assent/consent was obtained from each participant/legal guardian. Participants received £20 for each of the 1–1.5hr fMRI-Neurofeedback scan visit, and up to £10 for best performance during the session, as well as £20 for the post-training neuropsychological assessment, in total up to £150. They were also reimbursed for travel expenses (for further details see (Alegria et al., 2017)).

### 2.2 fMRI neurofeedback protocol

Boys were offered 14 fMRI-Neurofeedback runs (8.5min each) in four 1–1.5hr scan visits over 2 weeks. All data were motion corrected in real-time. The head coil used is relatively close fitting, and head motion was therefore inherently relatively difficult. In addition, chin straps and head pads were used to further minimise movement. This together with the motion correction built into both the real-time processing software (AFNI(http://afni.nimh.nih.gov/afni/about/)) and our offline processing package XBAM (http://www.brainmap.co.uk/xbam.htm) was sufficient to deal with potential motion problems. Each fMRI-Neurofeedback run consisted of seven rest (30s) and six activation (50s) blocks, starting with a rest block showing an underwater dolphin image, while activation blocks showed a video-clip of a rocket. Boys were asked to move the rocket towards space by any means they found helpful. Instructions were minimal (i.e., “you can try to concentrate on the rocket” or “try any other method that works for you”) as this has been shown to be more effective than explicit instructions (Sulzer et al., 2013) and instruction-free approaches are common in EEG-Neurofeedback for ADHD children (Gevensleben et al., 2014; Strehl et al., 2006). They received continuous feedback (every repetition time (TR), i.e., 2 s), about their brain activation in their target region of interest (ROI) via the rocket video-clip, with the direction and distance travelled in space proportional to their BOLD response. To enhance motivation, a score (0–10), reflecting the percentage of distance travelled through space during each run, appeared on the screen (e.g., 6 for 60%) and a monetary incentive (e.g., £6 for 6/60%) corresponding to the best performance in the session was given after the scan. Between runs (a few minutes' break), researchers briefly acknowledged participants' efforts in not moving their head, reminded them to keep doing so, and congratulated them for the score they obtained.

Between visits, boys had to practice daily brain self-regulation using a cue card depicting the video-clip rocket. After the last fMRI-Neurofeedback run, a 5-min fMRI transfer run was conducted. This was identical to the Neurofeedback training runs, using the same stimuli, but without the feedback (the rocket did not move), consisting of four rest and three activation blocks. Transfer runs measure retention of learning and are considered a proximal measure of successful transfer of training strategies to everyday life (Drechsler et al., 2007) (see (Alegria et al., 2017)).

### 2.3 Clinical outcome measures

The primary outcome measure was the ADHD Rating Scale (ADHD-RS-IV), a standard tool to assess ADHD symptoms according to DSM-5 and to monitor treatment effects (Dupaul et al., 1998), and the secondary outcome measure was the CPRS-R ADHD index (Conners et al., 1998), both rated by parents. ADHD-related difficulties and functional impairments were assessed with the Weekly Parent Ratings of Evening and Morning Behaviour-Revised scale (Wehmeier et al., 2009) and the Columbia Impairment Scale-Parent version (Bird et al., 1993), respectively.

### 2.4 fMRI-neurofeedback data acquisition and processing

Gradient-echo echo planar MR imaging and structural data were acquired on a 3T General Electric MR750 MRI scanner with a 12-channel head coil at the Centre for Neuroimaging Sciences, King's College London. The body coil was used for radiofrequency transmission. A T1-weighted structural scan (TR (repetition time)/TE (echo time) = 7.312/3.016 ms, flip angle = 11°, 196 × 1.2 mm slices, matrix size 256 × 256, 27 cm FOV, voxel size = 1.05 × 1.05 × 1.2 mm^3^), used as structural localizer, was collected at the beginning of each scanning session. fMRI-Neurofeedback scans were collected using an T2*-weighted gradient echo, echo-planar image sequence (TR/TE = 2000/30 ms, flip angle = 75°, 40 × 3 mm slices with a 0.3 mm slice gap, matrix size 64 × 64, 21.1 cm FOV, voxel size = 3.3 × 3.3 × 3.3 mm^3^). A whole-brain higher resolution gradient echo, echo-planar image scan for standard space normalization of individual activation maps was also acquired in the intercommissural plane with TR/TE = 3.000/30 ms, flip angle = 90°, 43 slices, slice thickness = 3.0 mm with a 0.3 mm slice gap, matrix size 128 × 128, 21.1 cm FOV, voxel size = 1.65 × 1.65 × 3.3 mm^3^.

A custom fMRI-Neurofeedback interface system (Bodurka and Bandettini, 2008) and the AFNI software (Cox, 1996) were used for real-time transfer and analysis of the fMRI data. The fMRI-Neurofeedback interface system ran on the scanner hardware to access the fMRI scans as they were reconstructed. The images were then transferred to an external Linux workstation where they were pre-processed using AFNI, a software with built-in real-time capacities. The effects of head motion were corrected for in real-time by the AFNI software, displaying running graphs of the motion parameters on the screen. We used the CA_N27_ML/TT_N template to define the target ROIs (rIFC) in AFNI structurally for each adolescent before each fMRI-Neurofeedback session. The ROI included the pars triangularis (14,138 voxels in the Talairach space of the template and 385 voxels when mapped to fMRI space) and the pars orbitalis (11,484 voxels in the Talairach space of the template and 308 voxels when mapped to fMRI space). A customized AFNI script automatically created a native-space image mask of the rIFC and the white matter (used as reference region, to cancel out non-specific global brain effects) based on the T1-weighted structural image and a two-volume echo-planar image localizer image matched to the fMRI sequence for the geometric distortion inherent in echo-planar image acquisitions (and also used as realignment target).

The image mask of the pre-selected ROIs was applied to the pre-processed fMRI images to extract in real-time the mean BOLD signal from each ROI. For each newly acquired brain volume, AFNI calculated a new set of values for each ROI, which were fed to a locally written program that generated a dynamic visual feedback display by means of the moving rocket. The threshold required for the rocket to move up was continuously updated based on past performance ((rIFC-white matter)-(rIFC_Previous_-white matter_Previous_)), where rIFC_Previous_ and white matter_Previous_ are the average activation of rIFC and white matter in the previous rest block. Participants were informed/reminded of the Neurofeedback delay (∼6s), caused by hemodynamic delay and data processing time, before each fMRI-Neurofeedback run.

### 2.5 Data analyses

#### 2.5.1 Clinical data

Some parents did not fill in all questionnaires. Missing data (<5%) were assumed to be completely at random. Multiple (i.e., 20) imputations were used for missing pre- and post-treatment data. The individual estimates from the multiply-imputed datasets were then used to calculate a combined estimate by applying Rubin's Rules (Little and Rubin, 2002). Repeated-measures mixed ANOVAs tested pre- and post-fMRI-Neurofeedback effects on clinical measures. Effect size (Cohen's d) was calculated as the difference between the means (pre-post) divided by the corresponding pooled standard deviation. Two-tailed Pearson correlation analyses tested correlations between fMRI-Neurofeedback induced connectivity changes (i.e., post-treatment functional connectivity – pre-treatment functional connectivity) and primary and secondary clinical outcome changes (i.e., post-treatment – pre-treatment clinical measures).

#### 2.5.2 fMRI-neurofeedback data

All 18 participants of the rIFC-Neurofeedback group (and 13 control boys) were included in the fMRI-Neurofeedback functional connectivity data analyses. Due to the relative novelty of installing fMRI-Neurofeedback on one of our new 3T scanners, several technical problems occurred (e.g., scanner database delays, mask creation issues, network problems between the various components of the Neurofeedback pipeline (e.g., no data transfer from the scanner to the processing server, or ROI information was not transferred to the paradigm presentation software), resulting in lack of feedback for the participants, all of which caused loss of fMRI-Neurofeedback runs. Therefore, the average number of fMRI-Neurofeedback runs for both groups was 11, with only 30% of participants, 4 participants in the rIFC-Neurofeedback group and 6 participants in the lPHG-Neurofeedback control group, completing all 14 runs. There was no group difference in number of runs completed (F (df = 1,29) = 2, p = 0.4). Therefore, only runs completed by at least about 70% of the participants were included, which resulted in only the first 11 (or less) fMRI-Neurofeedback runs being analysed (i.e., runs 1–11, with the lowest number of runs being 6).

Data were analysed using the non-parametric XBAM software package (Brammer et al., 1997). XBAM's non-parametric approach overcomes many of the issues associated with parametric software packages (e.g., poor control of FWE-corrected false positive cluster-wise inference rates) (Bullmore et al., 1999b; Eklund et al., 2016).

##### 2.5.2.1 MRI-neurofeedback preprocessing

fMRI data were first processed to minimise motion related artefacts (Bullmore et al., 1999a). A 3D volume consisting of the average intensity at each voxel over the whole experiment was calculated and used as a template. The 3D image volume at each time point was then realigned to this template by computing the combination of rotations (around the x, y, and z axes) and translations (in x, y, and z) that maximized the correlation between the image intensities of the volume in question and the template (rigid body registration). After realignment, data were then smoothed using a Gaussian filter at 7.8 mm FWHM (full-width half-maximum) to improve the signal-to-noise ratio of the images.

##### 2.5.2.2 Head motion

For each participant, the absolute and relative mean displacement were calculated by backwards differences to the first volume (absolute mean) or the previous volume (relative mean). Two-sample t-tests were performed to test for potential group differences in motion parameters. Furthermore, to test for the impact of motion on the functional connectivity results, we conducted Pearson correlation analyses between the absolute and relative mean displacement values and the significant functional connectivity changes in each group.

##### 2.5.2.3 Statistical analyses of functional connectivity changes with the seed regions

**Fig. 1:**
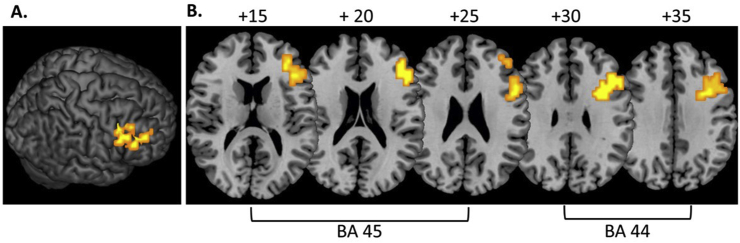
**Progressively increased activation in right inferior frontal cortex across 11 fMRI neurofeedback runs in 18 ADHD adolescents relative to a control group (N** = **13) who were trained to increase activation in left parahippocampal gyrus. A.** 3D image showing progressively increased rIFC activation. **B.** 2D axial slices showing progressively increased activation in two regions of right inferior frontal cortex (Brodmann area 45 and Brodmann area 44) (adapted from (Alegria et al., 2017)).

The previous study showed progressively increased activation with the number of fMRI-Neurofeedback runs in the rIFC-Neurofeedback group within two clusters of the rIFC: in Brodmann area 45 (peak Talairach coordinates (x; y;z); 43; 33; 16; p < 0.005; 47 voxels) and in Brodmann area 44 (peak Talairach coordinates (x; y;z), 36; 14; 29; p < 0.005; 75 voxels) (Alegria et al., 2017) (Fig. 1).

There were no differences between groups in the activation at baseline for Brodmann area 44 (t (df = 29) = −0.3, p = 0.113) or Brodmann area 45 (t (df = 29) = −1.7; p = 0.78).

Separate functional connectivity analyses were performed using these 2 clusters in Brodmann area 44 and Brodmann area 45 as seed regions. For each seed region, we extracted the average time series over the whole ROI for each subject in native space (reversing Talairach mapping). The average time series for each subject were then used as a model for a whole-brain correlation analysis producing functional connectivity maps. Functional connectivity maps for each subject were then transformed to standard space using a two-step procedure: first by rigid body transformation of the correlation data into a high-resolution echo-planar image of the same subject, and then by affine transformation onto a Talairach template (Talairach and Tournoux, 1988). Group connectivity maps were computed by determining the median correlation coefficient (across subjects) at each voxel. A median-based statistic was used across subjects in order to give more robustness against outlier effects.

Next, to examine whether there would be a positive or negative change in functional connectivity in the last relative to the first fMRI-Neurofeedback run with the two seed regions of rIFC, separate repeated-measures ANOVAs were conducted to test for functional connectivity differences with the seed regions (Brodmann area 44 and Brodmann area 45) between the last (11th or earlier) and first fMRI-Neurofeedback run. For this purpose, randomization-based tests for voxel- and cluster-wise differences were used as described in detail elsewhere (Bullmore et al., 1999b, 2001). For each of the analyses of positive or negative changes for each seed region, a voxel-level p < 0.05 was applied and a corresponding cluster-level statistical threshold was computed in order to obtain less than 1 false positive cluster per map for each analysis. Note that this resulted in different cluster-p-values in the different analyses of positive and negative functional connectivity changes with different seed regions.

In more detail, we used the cluster-level statistical analysis described by Bullmore et al (1999b) that was shown by extensive validation to give good type I error control at the cluster level. After setting the initial, voxel-level threshold to 0.05 to give maximum sensitivity and avoid type II errors, a second, cluster level threshold was computed for the resulting 3D voxel clusters using a data-driven based approach so that the final expected number of type I error clusters was less than one per whole brain. The necessary combination of voxel and cluster level thresholds is not assumed from theory but rather determined by direct permutation for each data set. In the current study an expected cluster-level type I error rate of <1 per brain was achieved by first applying a voxel-level threshold of 0.05 followed by thresholding the 3D clusters at various cluster-level thresholds computed from the datasets being considered. Readers unfamiliar with this method should be clear that a cluster level threshold of 0.05 (which resulted in less than 1 error cluster for the positive functional connectivity with the dorsal anterior cingulate cortex/caudate for example), which would be lenient in the context of the whole brain, was NOT applied to whole brain but rather to 3D clusters built from data previously thresholded at a voxel-wise level of 0.05.

### 2.6 Correlation analyses between functional connectivity changes and clinical and performance changes

Exploratory Pearson correlation analyses were conducted between functional connectivity change measures and the changes in clinical measures to test whether functional connectivity changes were associated with the clinical improvements observed in the rIFC-Neurofeedback group in the clinical ADHD symptom measures. For this purpose, we extracted for each subject the average correlation coefficient for each cluster that showed a positive or negative functional connectivity change with Brodmann area 44 or Brodmann area 45. Then the difference between functional connectivity post-Neurofeedback relative to before Neurofeedback in each cluster was correlated using two-tailed Pearson correlation analyses with the clinical changes (clinical measures post-Neurofeedback – clinical measures pre-Neurofeedback) in 2 key primary (ADHD-RS inattention, ADHD-RS hyperactivity/impulsiveness scores) and secondary clinical outcome measures (CPRS-R inattention, CPRS-R hyperactive/impulsive scores.

The primary and secondary change measures were correlated with each other with the exception of the changes in CPRS-R inattention score and ADHD-RS inattention score (r = 0.130; p = 0.485), the changes in CPRS-R inattention score and ADHD-RS hyperactivity/impulsiveness score (r = 0.025; p = 0.894), and the changes in CPRS-R hyperactivity/impulsivity score and ADHD-RS inattention score (r = 0.187; p = 0.313). Thus, the changes between the CRPS-R subscales (inattention and hyperactivity/impulsivity) were positively correlated (r = 0.415; p = 0.020) as were the changes between the ADHD-RS inattention and hyperactivity/impulsiveness subscales (r = 0.574; p = 0.001). The changes in the CRPS-R hyperactivity/impulsivity scores were positively correlated with the changes in the ADHD-RS hyperactivity/impulsiveness scores (r = 0.437; p = 0.014).

## 3 Results

### 3.1 Pre-post comparisons of outcome measures

The comparison between pre- and post-fMRI-Neurofeedback showed a significant decrease in both groups in the ADHD symptoms in all primary (ADHD-RS total scale, ADHD-RS Inattention subscale) and secondary outcome measures (CPRS-R ADHD-Index), with only a trend-wise significant reduction in ADHD-RS hyperactivity/impulsivity subscale in the rIFC-Neurofeedback group and a trend-wise improvement in the CPRS-R hyperactivity/impulsivity subscale in the control (lPHG-Neurofeedback) group.

Table 1 shows the clinical improvements in both groups in the primary and secondary outcome measures, the ADHD-RS and CPRS-R, respectively (for other measures see (Alegria et al., 2017)).

**Table 1:** Behaviour ratings before and after real-time fMRI Neurofeedback training for the rIFC-neurofeedback and lPHG-neurofeedback control ADHD groups. The primary and secondary outcome measures that were used for correlation analyses are printed in bold. ES d = effect size (Cohen's *d*); SD: Standard deviation.

	Pre-fMRI- neurofeedback	Post fMRI- neurofeedback	Pre-Post		
	Mean (SD)	Mean (SD)	F	*p*	ES *d*
**rIFC-neurofeedback group (N=18)**				F (1,17)	
**ADHD-Rating Scale**					
ADHD-RS total score	36.72 (9.43)	30.15 (11.63)	6.00	0.025	0.62
**ADHD-RS inattention**	19.83 (4.46)	15.94 (6.78)	6.38	0.022	0.68
**ADHD-RS hyperactivity/impulsivity**	16.89 (5.71)	14.21 (6.15)	3.82	0.067	0.45
**Conner's Parent Rating Scale (T-score)**					
*ADHD index*	13.61 (4.80)	10.67 (5.79)	5.29	0.034	0.55
Global index	84.06 (6.81)	76.42 (12.16)	8.91	0.008	0.78
**Inattention**	81.72 (7.20)	74.30 (9.19)	8.45	0.010	0.90
**Hyperactivity/impulsivity**	85.06 (9.56)	78.83 (14.42)	9.15	0.008	0.51
**lPHG-neurofeedback control group (N=13)**				F (1,12)	
**ADHD-Rating Scale**					
ADHD-RS total score	37.77 (11.39)	29.30 (10.95)	49.42	<0.001	0.76
**ADHD-RS inattention**	20.92 (4.59)	16.04 (6.28)	30.47	<0.001	0.89
**ADHD-RS hyperactivity/impulsivity**	16.85 (7.48)	13.26 (6.14)	16.35	0.002	0.52
**Conner's Parent Rating Scale (T-score)**					
*ADHD index*	16.46 (2.88)	11.90 (5.20)	18.63	0.001	1.08
Global index	87.31 (6.10)	80.64 (13.12)	6.30	0.027	0.65
**Inattention**	84.92 (5.81)	76.61 (10.89)	7.18	0.020	0.95
**Hyperactivity/impulsivity**	85.92 (9.28)	81.05 (13.04)	3.61	0.082	0.43

### 3.2 Functional connectivity results

#### 3.2.1 Head motion

For the rIFC-Neurofeedback group, the absolute mean displacement was 1.16 (SD = 0.78), and the relative mean displacement was 0.218 (SD = 0.19); for the lPHG-Neurofeedback (control) group the absolute mean displacement was 0.88 (SD = 0.57), and the relative mean displacement was 0.14 (SD = 0.15). T-test showed no significant group differences for absolute or relative mean displacements (absolute mean displacement: t (df = 29) = 1.076, p = 0.291; relative mean displacement: t (df = 29) = 1.190, p = 0.244).

There were no significant correlations in either group between the changes in functional connectivity and the absolute or relative mean displacements (for statistical details see Supplementary Table S1).

#### 3.2.2 Positive and negative functional connectivity changes using the clusters in Brodmann area 45 and Brodmann area 44 as seed region

##### 3.2.2.1 Brodmann area 45 as seed region

For the ANOVA of positive functional connectivity changes between the last relative to the first fMRI-Neurofeedback run with Brodmann area 45, less than one false positive cluster was observed with voxel-wise p < 0.05 and cluster p < 0.02. Increased functional connectivity was observed between rIFC and a cluster in right caudate/anterior cingulate cortex. For the ANOVA of decreased functional connectivity with Brodmann area 45 for the last relative to the first fMRI-Neurofeedback run, less than one error cluster was observed with voxel-wise p < 0.05 and cluster p < 0.002.

**Fig. 2:**
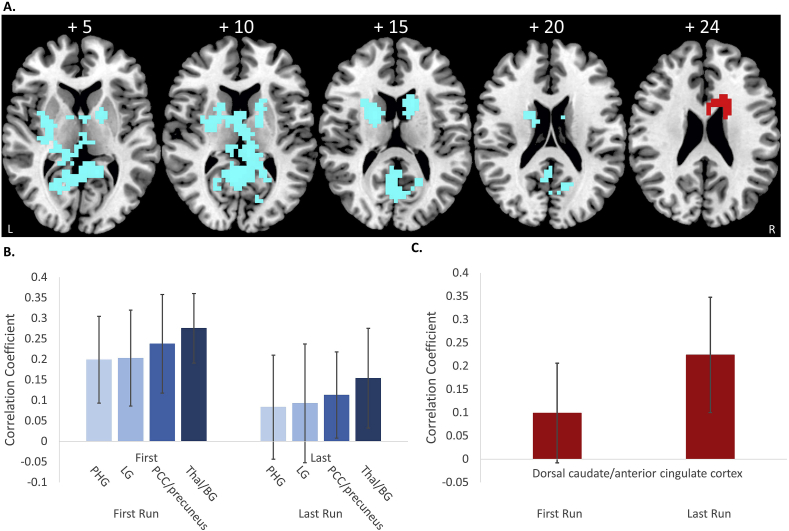
**Results of the functional connectivity analysis using the cluster in Brodmann area 45 as seed region**. A. Axial slices of positive (red) and negative (blue) changes in functional connectivity with the cluster in Brodmann area 45 as seed region. B. Average correlation coefficient values for the 4 regions (PHG = parahippocampal gyrus; LG = lingual gyrus; PCC/precuneus = posterior cingulate gyrus; Thal/BG = thalamus/basal ganglia) that showed a decrease in functional connectivity for the last > first run are shown for the first and the last run. (C.) Average correlation coefficients of the dorsal caudate/anterior cingulate cluster that showed a positive functional connectivity change are shown for the first and last run.

Decreased functional connectivity was found between rIFC and 4 posterior clusters comprising left parahippocampal gyrus/hippocampus/thalamus/putamen/insula; left lingual gyrus; bilateral posterior cingulate cortex/precuneus/calcarine gyrus; and thalamus, ventral basal ganglia and insula (Fig. 2 and Table 2A).

**Table 2:** Changes in positive and negative functional connectivity with the two seed regions of rIFC.

Brain regions	Brodmann area	Peak Talairach coordinates (x; y;z)	Cluster size (voxels)	Cluster p-value
**A. Seed region Brodmann area 45**				
*Increased connectivity*				
**R dorsal caudate/anterior cingulate cortex**^a^	**24/32**	**22; 15; 20**	**30**	**0.02**
*Decreased connectivity*				
L parahippocampal gyrus/hippocampus/thalamus/putamen/insula	27/30/36	−18.; −30; −3	75	0.012
L lingual gyrus	18	−18; −56; 3	25	0.015
**BL PCC/precuneus/calcarine gyrus**^a^	**30/29/23/31/17/18/19**	**4; -45; 7**	**192**	**0.008**
BL thalamus/ventral caudate/putamen/L insula/R pallidum		0; 0; 10	286	0.009

**B. Seed region Brodmann area 44**				
*Increased connectivity*				
**R dorsal anterior cingulate cortex**^a^	**24**	**18; -15; 33**	**21**	**0.041**
*Decreased connectivity*				
**BL PCC/precuneus/hippocampus/parahippocampal gyrus/thalamus/calcarine/lingual gyrus**^a^	**27/30/29/26/27/17/27/18**	**7; -41; 7**	**224**	**0.0006**

a Areas in bold are regions where the connectivity changes were significantly different to the control group. However, these were not corrected for multiple testing.BL: bilateral, L: left, R: right.

To test whether the functional connectivity changes which we observed with the seed regions in Brodmann area 44 and Brodmann area 45 in the rIFC-Neurofeedback group, were specific to the rIFC-Neurofeedback group, we tested whether these functional connectivity changes were significantly different between groups using a repeated measures ANOVA. For this purpose, we extracted for the lPHG-Neurofeedback control group all the correlation coefficients for the first and last run in all clusters that showed changes in functional connectivity with Brodmann area 44 and Brodmann area 45 in the active group. Then we conducted repeated measures ANOVAs to compare the changes in functional connectivity between the last and first run between groups (with run as within-group repeated measure and group as between-group measure).

Significant group differences were observed for the cluster in the anterior cingulate/caudate that showed a positive connectivity change with Brodmann area 45 (F (df = 1, 29) = 4; p < 0.05). From the 4 clusters that showed negative functional connectivity changes with Brodmann area 45, significant group effects were only observed in the cluster in posterior cingulate/precuneus that showed significant decrease in functional connectivity in the rIFC-Neurofeedback group compared to the control group with neurofeedback (F (df = 1, 29) = 8, p < 0.007). The clusters in lingual gyrus (F (df = 1,29) = 3, p < 0.08) and in bilateral thalamus/ventral basal ganglia (F (df = 1,29) = 3, p < 0.09) only showed a trend-level difference of being decreased in the rIFC-Neurofeedback group relative to controls, while the cluster in left parahippocampal gyrus and hippocampus was not significantly different between groups (F (df = 1,29) = 3, p < 0.113).

##### 3.2.2.2 Brodmann area 44 as seed region

**Fig. 3:**
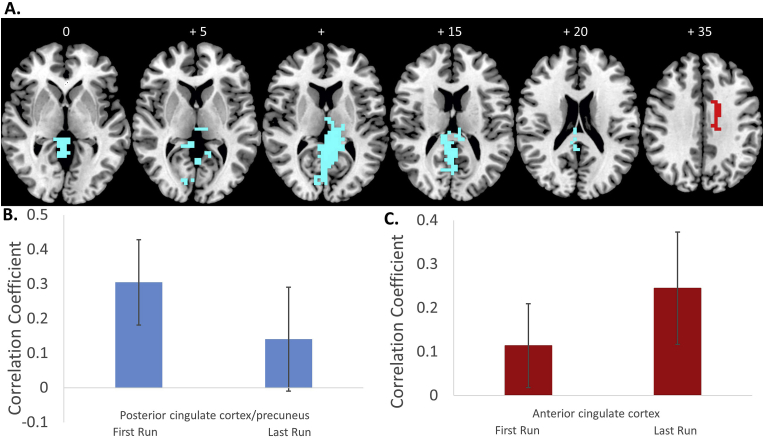
**Results of the functional connectivity analysis using the cluster in Brodmann area 44 as seed region. A.** Axial slices of positive (red) and negative (blue) changes in functional connectivity with the cluster in Brodmann area 44 as seed region. **B**. Average correlation coefficient values for the cluster in posterior cingulate/precuneus that showed a decrease in functional connectivity with Brodmann 44 for the last > first run are shown for the first and the last run. **C.** Average correlation coefficients of the dorsal anterior cingulate cortex cluster that showed a positive functional connectivity change with Brodmann area 44 are shown for the first and last run.

For the ANOVA of positive functional connectivity changes between the last relative to the first fMRI-Neurofeedback run in Brodmann area 44, less than one false positive cluster was observed with voxel-wise p < 0.05 and cluster p < 0.05. This revealed increased functional connectivity with the right anterior cingulate cortex. For the ANOVA of negative functional connectivity changes between the last relative to the first fMRI-Neurofeedback run in Brodmann area 44, less than one error cluster was observed with voxel-wise p < 0.05 and cluster p < 0.001. Decreased functional connectivity was observed in a large cluster comprising the bilateral precuneus/posterior cingulate cortex/hippocampus/parahippocampal gyrus/thalamus/lingual gyrus (Fig. 3 and Table 2B).

Significant group differences were observed for both the cluster in the anterior cingulate (F (df = 1,29) = 4; p < 0.05), that showed significant increase in functional connectivity in the rIFC-Neurofeedback group compared to the control group, and the cluster in posterior cingulate cortex/precuneus that showed significant decrease in functional connectivity in the rIFC-Neurofeedback group compared to the lPHG-Neurofeedback control group (F (df = 1,29) = 12; p < 0.001).

The positive functional connectivity changes we observed in the rIFC-Neurofeedback group between Brodmann area 44 and Brodmann area 45 and the anterior cingulate and dorsal caudate/anterior cingulate, respectively, and the negative functional connectivity changes between 44 and Brodmann area 45 and the posterior cingulate/precuneus were hence specific to the rIFC-Neurofeedback group as they were significantly different from the functional connectivity changes in these regions in the control group.

### 3.3 Correlations between positive and negative functional connectivity changes between the two seed clusters in rIFC and resulting clusters, and clinical outcome changes

**Fig. 4:**
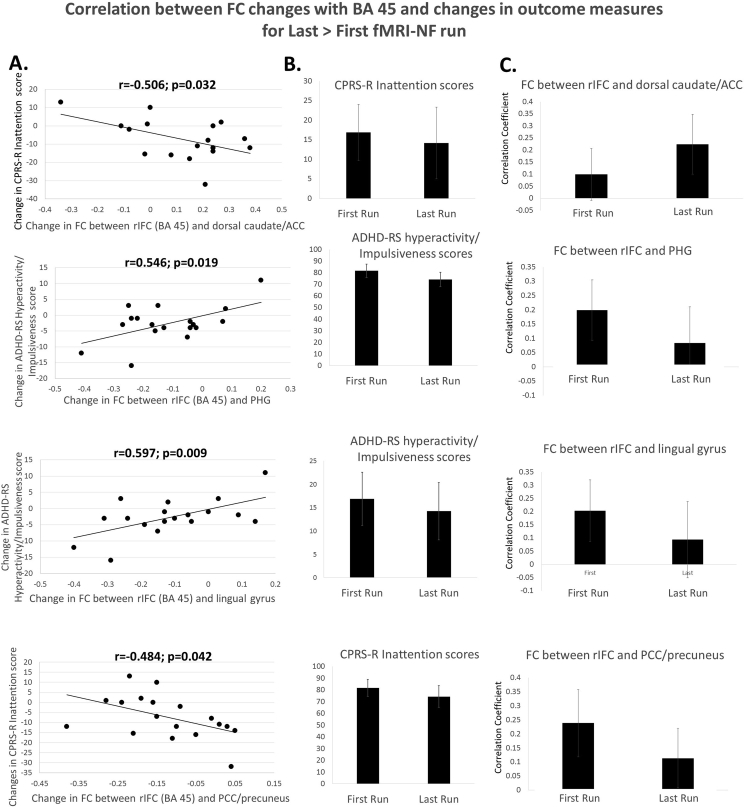
**Correlations between functional connectivity changes with Brodmann area 45 and changes in outcome measures for Last > First run. A.** Correlations between positive and negative functional connectivity changes between the seed cluster in rIFC Brodmann area 45 and the 4 resulting clusters (PHG = parahippocampal gyrus; LG = lingual gyrus; PCC/precuneus = posterior cingulate gyrus; Thal/BG = thalamus/basal ganglia) and clinical outcome changes. **B.** Clinical outcome measures for the first and last run. **C.** Correlation coefficients for the functional connectivity of the 4 clusters with the seed region in Brodmann area 45 for the first and the last fMRI-NF runs. Note that correlations were not adjusted for multiple testing.

To test whether the resulting clusters that showed positive and negative functional connectivity changes with the two seed regions of rIFC were correlated with behavioural improvements, average correlation coefficients were extracted for each subject in regions where changes in functional connectivity with the rIFC were found. Exploratory two-tailed Pearson correlation analyses were then performed with changes in primary and secondary clinical outcome measures post-prefMRI-Neurofeedback (see Table 3). Increased positive functional connectivity between the rIFC (Brodmann area 45) and dorsal caudate/anterior cingulate cortex was significantly negatively correlated with reduced CPRS-R Inattentive score (r = −0.5, p = 0.032), suggesting that improved functional connectivity between IFC and dorsal caudate/anterior cingulate cortex was associated with reduced CPRS-R inattention symptom scores (Fig. 4). Decreased functional connectivity between rIFC (Brodmann area 45) and PHG and between rIFC and lingual gyrus showed positive correlations with reduced ADHD-RS hyperactivity/impulsiveness scores (PHG: r = 0.6, p = 0.019; lingual gyrus: r = 0.6, p = 0.009). Decreased functional connectivity between the rIFC (Brodmann area 45) and posterior cingulate cortex/precuneus was negatively correlated with reduced CPRS-R inattentive scores (r = −5, p = 0.042) (Fig. 4).

**Table 3:**
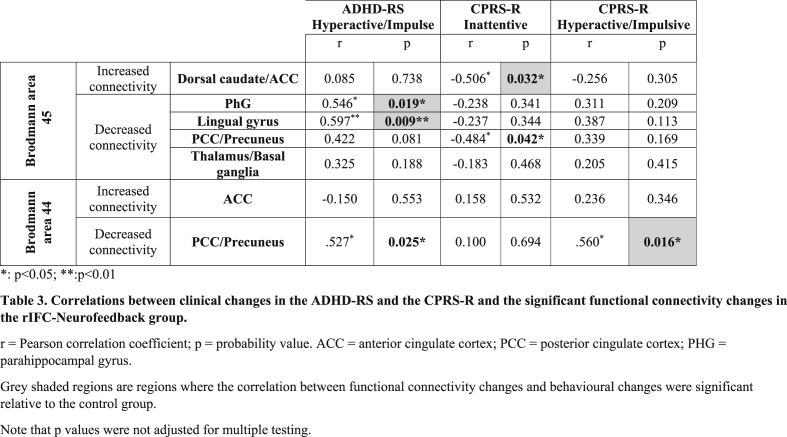
Correlations between clinical changes in the ADHD-RS and the CPRS-R and the significant functional connectivity changes in the rIFC-Neurofeedback group.

**Fig. 5:**
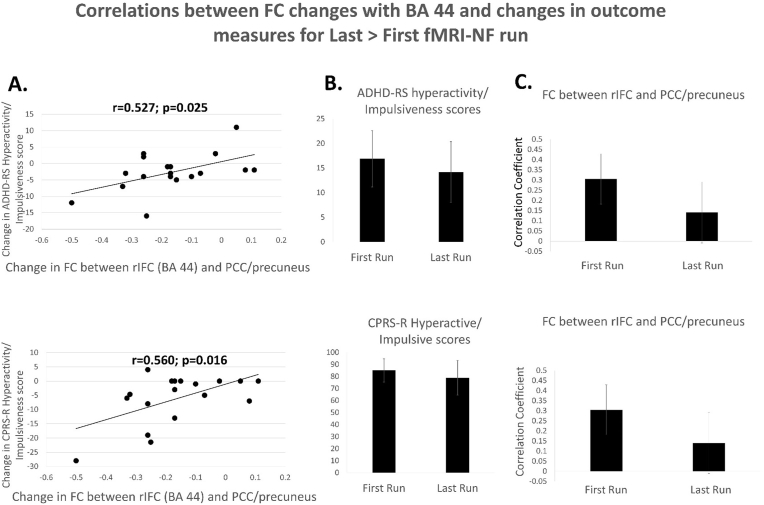
**Correlations between functional connectivity changes with Brodmann area 44 and changes in outcome measures for Last > First run. A.** Correlations between positive and negative functional connectivity changes between the seed cluster in rIFC Brodmann area 44 and the cluster in posterior cingulate cortex (PCC)/precuneus, and clinical outcome changes. **B.** Clinical outcome measures for the first and last run. **C.** Correlation coefficients for the functional connectivity of PCC/precuneus with the seed region in Brodmann area 44 for the first and the last fMRI-NF runs. Note that correlations were not adjusted for multiple testing.

Increased negative functional connectivity between the rIFC (Brodmann area 44) and posterior cingulate cortex/precuneus were positively correlated with the reduction in the hyperactive/impulsive scores in both the ADHD-RS and the CPRS-R (ADHD-RS hyperactivity/impulsiveness score (r = 0.5, p = 0.025); CPRS-R hyperactive/impulsive score: r = 0.6, p = 0.016) (Fig. 5). (see also Table 3).

To test for specificity of these associations, we also tested for correlations between the functional connectivity with these clusters and clinical symptom measures in the control group. None of the functional connectivity changes in any of the above mentioned clusters showed significant correlations with the clinical measures (ADHD-RS and CPRS-R) in the control group with the exception of the functional connectivity changes between Brodmann area 45 and the anterior cingulate/caudate that was positively correlated with the CPRS-R inattention subscore (r = 0.6, p < 0.02) (while negatively in the rIFC-neurofeedback group) and between Brodmann area 45 and Brodmann area 44 and the respective posterior cingulate/precuneus clusters that were negatively correlated with changes in the CPRS-R inattention scores (for Brodmann area 44; r = −0.6, p < 0.04; for Brodmann area 45: r = −0.6, p < 0.023). Direct group comparisons of the correlations showed that the difference in correlations between the change in functional connectivity between Brodmann area 45 and anterior cingulate/caudate and the change in CPRS-R inattention scores was highly significant as it was negative in the rIFC-Neurofeedback group (associated with symptom improvement) and positive in the lPHG-Neurofeedback control group (associated with symptom deterioration) (z = −3, p < 0.001). The group difference in correlation between the change in functional connectivity between Brodmann area 44 and the posterior cingulate/precuneus and the change in CPRS-R hyperactive/impulsive score was also significant (controls r = 0.001; p = 0.9) (z = 1.7; p < 0.045). Further significant were the group differences in correlation between the changes in ADHD-RS hyperactivity/impulsive scores and the functional connectivity changes between Brodmann area 45 and the parahippocampal gyrus (z = 2.7, p < 0.003) which in controls were associated with worse clinical changes (controls r = −0.4, p = 0.9); and the functional connectivity changes between Brodmann area 45 and lingual gyrus (z = 2.6, p < 0.005) which were also associated with worse clinical outcomes in controls (controls: r = −0.35, p = 0.24); while both were associated with better clinical changes in the rIFC-Neurofeedback group.

### 3.4 Functional connectivity changes in the lPHG-Neurofeedback control group

We also tested for functional connectivity changes for the last > first run in the control group who showed 3 regions to be progressively more activated across the 11 fMRI-Neurofeedback runs relative to the rIFC-Neurofeedback group in areas of the lPHG, in Brodmann area 36, Brodmann area 35 and Brodmann area 30, using the same methods as described above (see Fig. S2, supplementary material).

For Brodmann area 36, there was no change in positive functional connectivity, but an increase in negative functional connectivity (ANOVA, voxel p < 0.05, cluster p < 0.004) between the last > first run with the cerebellum which extended into the parahippocampal gyrus and brain stem (see Fig. S2A, Table S2A in supplementary material).

For Brodmann area 35, there was also no positive functional connectivity change, but an increase in negative functional connectivity (ANOVA, voxel-wise p < 0.05, cluster-wise p < 0.009) for last > first run with right anterior cingulate gyrus (see Fig. S2B, Table S2B in supplementary material).

For Brodmann area 30, there was no negative functional connectivity change, but an increase in positive functional connectivity (ANOVA, voxel-wise p < 0.05, cluster p < 0.01) for last > first run with precuneus/paracentral gyrus extending into the superior parietal lobe and the supplementary motor area (see Fig. S2C, Table S2C in supplementary material).

None of the functional connectivity changes overlapped with any of the functional connectivity changes in the rIFC-Neurofeedback group. If anything, the increase in negative functional connectivity between Brodmann area 35 and dorsal anterior cingulate for last > first run was in the opposite direction to the increase of positive functional connectivity between rIFC (Brodmann area 45) and dorsal anterior cingulate in the rIFC-Neurofeedback group and the increase in positive functional connectivity between Brodmann area 30 and the posterior cingulate/precuneus for last > first run is in the opposite direction to the decrease in functional connectivity between rIFC (Brodmann area s 45 and 44) and posterior cingulate cortex in the rIFC-Neurofeedback group.

We also tested for correlations between the functional connectivity changes and the clinical symptom changes in the ADHD-RS and the CPRS-R. No significant correlations were observed (see Supplementary Table S3).

## 4 Discussion

### 4.1 Summary of the main findings

In this study we tested whether upregulation of a specific frontal region, i.e. rIFC, with fMRI-Neurofeedback in ADHD adolescents is associated with changes in functional connectivity between rIFC and other brain regions using seed-based whole brain functional connectivity analyses. We found that rIFC upregulation after 11 runs of 8.5 min of fMRI-Neurofeedback was associated with increased functional connectivity with anterior cingulate and dorsal caudate and with decreased functional connectivity with posterior regions of the DMN including posterior cingulate, precuneus, parahippocampal and lingual gyri. Furthermore, both the positive functional connectivity changes with anterior cingulate and dorsal caudate and the negative functional connectivity changes with areas of the DMN were significantly associated with ADHD symptom improvements after to relative before the fMRI-Neurofeedback training. In addition, the findings were specific to the rIFC-Neurofeedback group as most of the functional connectivity changes and most correlation associations with symptom changes significantly different when compared to the lPHG-Neurofeedback control group who did not show these changes nor correlations of these changes with clinical symptom improvements. The study shows for the first time, that the upregulation of a specific frontal region in ADHD adolescents with fMRI-Neurofeedback is associated with underlying ***neural network*** changes that are linked with clinical improvements and that these are specific to the frontal upregulation.

### 4.2 Increased functional connectivity between rIFC and dorsal anterior cingulate cortex and caudate

The increased functional connectivity of rIFC with areas of the cognitive control network, comprising rIFC, anterior cingulate cortex and the dorsal caudate (Guo et al., 2018; He et al., 2009, Niendam et al., 2012) was hypothesised. rIFC is a key cognitive control hub region that has been associated with inhibitory control, sustained attention, visual-spatial working memory, cognitive switching and even time estimation (de la Vega et al., 2017; Hugdahl et al., 2015; Niendam et al., 2012; Radua et al., 2014), all of which are functions found to be consistently impaired in ADHD (Noreika et al., 2013; Rubia et al., 2014a; Willcutt et al., 2005). rIFC forms part of the cognitive control network that mediates motor and cognitive inhibition, cognitive switching and working memory, and that includes the dorsolateral prefrontal cortex, the dorsal anterior cingulate cortex, the supplementary motor area, the dorsal caudate, anterior insula and inferior parietal regions (Cole and Schneider, 2007; de la Vega et al., 2017; Dixon et al., 2018; Guo et al., 2018; Kim et al., 2012; Hugdahl et al., 2015, Niendam et al., 2012). rIFC is also closely connected and typically coactivated with dorsal caudate and anterior cingulate cortex for “cognitive control” in the Neurosynth fMRI database (www.neurosynth.org) (Yarkoni et al., 2011). This IFC-cingulo-striatal cognitive control network has been found to be consistently underactivated in ADHD patients in several fMRI meta-analyses of cognitive control (Cortese et al., 2012; Hart et al., 2012, 2013; Lei et al., 2015; McCarthy et al., 2014; Norman et al., 2016). The finding of increased functional connectivity of rIFC with the dorsal caudate and the anterior cingulate cortex after upregulation training of rIFC in ADHD adolescents hence suggests that fMRI-Neurofeedback of rIFC has not only resulted in increased strength of activation in this specific frontal region, but also in increased strength of functional connectivity within a rIFC-striato-cingulate ***cognitive control network.*** Furthermore, we found that the strengthened neural network connectivity between rIFC and striato-cingulate cognitive control regions was associated with improved ADHD inattention symptoms and that most of the connectivity changes and the association with attention symptom improvement were specific to the rIFC-Neurofeedback group. The findings have important implications for the fMRI-Neurofeedback neurotherapy field as they suggest that patients with neural network difficulties may benefit from neurofeedback of one dominant (i.e., usually frontal) part of the network which in turn will lead to co-activation of entire networks connected to the target region. It suggests that in addition to targeting deficient networks with functional connectivity-based neurofeedback (see McDonald et al., 2017; Yamashita et al., 2017), it is possible to indirectly enhance neural networks by targeting the fronto-dominant part of this network.

Several fMRI-Neurofeedback studies observed changes in functional connectivity associated with fMRI-Neurofeedback training of a frontal brain region with other cortical and subcortical regions in healthy adults and other clinical groups. Thus, in healthy adults, rIFC upregulation led to increased functional connectivity with several other prefrontal regions (Rota et al., 2011), supplementary motor area upregulation training increased functional connectivity with middle, superior and anterior cingulate cortex regions (Sepulveda et al., 2016), left lateral prefrontal upregulation led to increased functional connectivity with right prefrontal and posterior cingulate regions (Sarkheil et al., 2015) and increased self-regulation of the left dorsolateral prefrontal cortex was associated with increased functional connectivity with striato-thalamic, inferior parietal and cerebellar regions (Zhang et al., 2015a, 2015b). Inversely, self-regulation of subcortical regions has been shown to increase functional connectivity with cortical regions in healthy subjects and in other clinical groups. For example, self-regulation of the insula or the amygdala in the context of aversive stimuli was associated with increased functional connectivity with medial and lateral frontal regions in healthy subjects (Paret et al., 2016b; Veit et al., 2012), in patients with schizophrenia (Ruiz et al., 2013) and with bipolar disorder (Paret et al., 2016a) and with inferior frontal cortex, temporal regions and precuneus in patients with depression, which was furthermore associated with improved clinical symptoms (Young et al., 2018).

Some fMRI-Neurofeedback studies in healthy controls or in other disorders furthermore observed changes in intrinsic functional connectivity in resting state fMRI scans before and after fMRI-Neurofeedback training, beyond the timeframe of the fMRI-Neurofeedback training, a day or even a week later. For example, in Huntington's disease, fronto-striatal functional connectivity was increased after fMRI-Neurofeedback of the supplementary motor area (Papoutsi et al., 2018). In people with subclinical anxiety, self-regulation of the bilateral orbitofrontal cortex led to increased functional connectivity with dorsolateral prefrontal regions of cognitive control but to reduced functional connectivity with limbic regions including amygdala, hippocampus, thalamus and insula; these persisted several days after the training and were furthermore associated with improved anxiety symptoms (Scheinost et al., 2013). Other studies observed that upregulation of the amygdala with fMRI-Neurofeedback in major depression was associated with increased functional connectivity in several temporal and frontal regions, including inferior frontal and dorsal anterior cingulate cortex areas (Young et al., 2018; Yuan et al., 2014), while amygdala downregulation with fMRI-Neurofeedback was associated with increased functional connectivity of the amygdala with ventromedial, anterior cingulate and dorsolateral prefrontal regions in post-traumatic stress disorder (Nicholson et al., 2017), borderline personality disorder (Paret et al., 2016a) or healthy adults (Zotev et al., 2013).

In conclusion, there is consistent evidence that the self-regulation training of an isolated brain region leads to a strengthening of functional connectivity with other brain regions that form part of the networks associated with the target brain region. The findings of this study expand the existing literature by showing for the first time increased fronto-striato-cingulate functional connectivity after rIFC upregulation in a pediatric ADHD population, which was furthermore associated with improved attention symptoms. The specificity of the functional connectivity change in this fronto-striato-cingulate network and of its association with inattention symptom improvement relative to the control group further strengthens the finding by showing that it is specific to the upregulation training of rIFC.

### 4.3 Negative functional connectivity of rIFC with areas of the DMN

To our knowledge, this is the first study to show that the upregulation of a frontal region not only leads to increased functional connectivity with related regions of the same network, but also to reduced functional connectivity with regions of the DMN and that this is furthermore associated with an improvement in clinical ADHD symptoms. The negative functional connectivity changes between rIFC and posterior areas of the DMN in ADHD are hence particularly interesting. The DMN has been associated with task-irrelevant thinking and with mind-wandering (Christoff et al., 2016; Fox et al., 2005; Raichle, 2015; Raichle et al., 2001). The DMN is progressively more deactivated during progressively more challenging tasks in healthy adults and children and this has been associated with a necessary reduction of mind-wandering during task performance (Christoff et al., 2016; Sato et al., 2014) as also shown in a parametric association between DMN activity and mind-wandering (Mason et al., 2007). Abnormal deactivation of the DMN has been associated with increased attentional lapses in both children and adults (Phillips et al., 2015; Sato et al., 2014). The DMN has been a direct target of fMRI-Neurofeedback in healthy subjects who have been shown to be able to self-regulate the DMN network after the training (McDonald et al., 2017).

rIFC activation increases in ADHD adolescents after relative to before fMRI-Neurofeedback in both Brodmann area 45 and Brodmann area 44 were associated with decreased activation in clusters that included typical posterior DMN regions such as posterior cingulate, precuneus, parahippocampal and lingual gyri (Christoff et al., 2016; Fox et al., 2005; Raichle, 2015; Raichle et al., 2001); however, only the changes in the key DMN regions of posterior cingulate and precuneus were specific to the rIFC-Neurofeedback group when compared to the lPHG-Neurofeedback control group.

Interestingly, the posterior cingulate cortex clusters also extended to posterior thalamus and a more ventral striatal region relative to the dorsal striatal region that was increased in functional connectivity. Although not considered classical DMN regions, posterior thalamus and striatum form part of the DMN in the automatic fMRI meta-analyses generated in the neurosynth database under the search term: “default network” (www.neurosynth.org) (Yarkoni et al., 2011). Furthermore, children and adolescents have an immature DMN, and a recent meta-analysis of the DMN in children includes the thalamus, striatum and posterior insula (Mak et al., 2017). The findings hence suggest that upregulation of rIFC with fMRI-Neurofeedback may elicit downregulation of the DMN that is typically anti-correlated with task-positive rIFC networks in children and adults (Cole et al., 2014; Fox et al., 2005; Mason et al., 2007; Raichle, 2015; Raichle et al., 2001; Sripada et al., 2014a). This hypothesis of a strengthening of task-positive cognitive control networks and a weakening of the association between rIFC and the DMN after self-upregulation training of rIFC in ADHD is furthermore backed up by the dissociated correlation findings. They showed that both the positive functional connectivity changes with dorsal caudate/anterior cingulate cortex as well as the negative functional connectivity changes with posterior regions of the DMN were associated with clinical ADHD symptom improvements. Thus, inattention symptom scores in the CPRS-R were negatively correlated with the positive functional connectivity changes in rIFC-striato-cingulate networks and inattention scores on the CPRS-R as well as hyperactivity/impulsiveness scores in the ADHD-RS and the CPRS-R were positively correlated with the negative functional connectivity changes of rIFC with posterior DMN regions (Fig. 4, Fig. 5). This suggests that both the functional connectivity strengthening in the rIFC-striato-cingulate network as well as the weakening of functional connectivity between rIFC and posterior DMN regions were associated with improvements in ADHD symptoms. Furthermore, the functional connectivity changes between Brodmann area 44 and Brodmann area 45 and regions of the DMN in posterior cingulate and precuneus and their associations with symptom improvements were specific to the rIFC-Neurofeedback group. To our knowledge, only one fMRI–Neurofeedback study has directly targeted the DMN by teaching down-regulation of the posterior cingulate cortex, which resulted in downregulation of other functionally interconnected DMN regions such as medial prefrontal cortex and anterior cingulate cortex (Zhang et al., 2015a).

An alternative explanation for decreased functional connectivity with striato-thalamic regions could be that neurofeedback allows subjects to develop greater conscious control over activity in their rIFC by reducing the influence of cortico-striato-thalamocortical loops on the region. rIFC is closely interconnected with striato-thalamic regions forming fronto-striato-thalamic networks of top-down control (Alexander and Crutcher, 1990; Arnsten and Rubia, 2012). A downregulation of striato-thalamic regions could hence suggest a neurofeedback-induced shift within the network toward enhanced rIFC activation in detriment to striato-thalamic components of the network. In line with this theory, similar effects of downregulation of more ventral striatal and right thalamic regions were observed in resting state fMRI data after fMRI-Neurofeedback of the supplementary motor area in healthy adults (Hampson et al., 2011).

The specificity of the findings of increased rIFC-striato-cingulate functional connectivity and decreased functional connectivity of rIFC with posterior DMN regions and their correlations with symptom improvements is interesting, in particular in view that most findings were in the opposite direction in the control group, were fMRI-Neurofeedback induced lPHG activation increase was associated with ***reduced*** functional connectivity with anterior cingulate cortex and with ***increased* functional connectivity** with areas of the DMN such as precuneus.

It is interesting to note, however, that the lPHG-Neurofeedback control group also improved in ADHD symptoms despite not showing the functional connectivity changes observed in the rIFC-Neurofeedback group and which were associated with symptom improvements in the rIFC-Neurofeedback but not the control group.

One potential explanation could be that the lPHG is more difficult to self-regulate than rIFC, maybe due to the fact that rIFC is a self-control region (Rae et al., 2014; Rubia et al., 2003). There is in fact evidence that higher-order association regions are easier to self-regulate than lower-level primary function regions (Harmelech et al., 2015). If the lPHG-Neurofeedback training was more challenging than the rIFG-Neurofeedback training, and demanded superior self-regulation skills, then the superior self-regulation effort and skill learning of the control group could potentially explain the comparable clinical improvements of both groups, despite lPHG not being a key deficit region for ADHD.

Although we trained the upregulation of the parahippocampal gyrus, that is associated with visual-spatial processing and episodic memory (Aminoff et al., 2013), we cannot exclude that the training may have affected nearby hippocampal regions. There is evidence that the interconnection of anterior hippocampal systems with frontal regions facilitates the incorporation of relevant prior experiences into executive function and hence play an important role in executive functions (Murty et al., 2016), which are typically associated with ADHD and poor self-control (Rubia, 2018a,b). The hippocampus also plays a critical role in integrating sensory information during learning and consolidation of memories (Cohen, 2015) and may hence play an important role in neurofeedback learning. Furthermore, the control group showed increased functional connectivity between parahippocampal region Brodmann area 30 and posterior parietal attention regions which are typically closely interconnected to parahippocampal gyrus and important for visual-spatial attention functions (Aminoff et al., 2013). Problems with attention functions underpinned by parietal abnormalities are highly relevant to ADHD (Hart et al., 2012, 2013, Rubia, 2018a,b) and the positive functional connectivity with parietal attention regions could hence also be related to clinical improvements in inattention.

It is possible that the lack of correlations between connectivity changes and behaviour were particularly underpowered in the smaller group. A placebo effect is also possible. We argue, however, that a placebo effect in the active group is unlikely given the fact that the connectivity changes with rIFC - which was the target of the neurofeedback treatment-correlated with the positive behavioural changes. While the placebo effect has also been associated with connectivity-behaviour associations, the regional location typically involves other regions such as ventromedial prefrontal cortex, insula, and limbic areas (Geuter et al., 2017). However, we cannot exclude potential placebo effects via non-tested brain mechanisms.

### 4.4 Key abnormalities in ADHD in the cognitive control and DMN networks

The findings that fMRI-Neurofeedback of rIFC in ADHD led to increased functional connectivity in fronto-striatal networks and decreased functional connectivity with posterior DMN regions and that this is associated with improvement in clinical ADHD symptoms are particularly interesting because poor activation of fronto-striatal systems and poor deactivation of the DMN are key functional deficits in ADHD and are associated with poor executive function performance (Rubia et al., 2014a, Rubia, 2018a,b). ADHD patients have consistent abnormalities in the deactivation of the DMN during cognitive tasks, as shown in fMRI meta-analyses (Cortese et al., 2012; Hart et al., 2012, 2013; Lei et al., 2015), and in individual fMRI studies of parametric task design where more difficult task conditions, unlike in healthy control boys, did not elicit increased deactivation of the DMN in ADHD patients nor did they increase fronto-striatal task-positive activation (Christakou et al., 2013; Fassbender et al., 2009; Salavert et al., 2015). Furthermore, the poor deactivation of DMN regions has been shown to be inversely associated with decreased fronto-striatal activation during attention and inhibition tasks and to be associated with worse task performance (Christakou et al., 2013; Liddle et al., 2011; van Rooij et al., 2015). This has also been observed in a large connectomic multi-site resting state analysis (ADHD200) where 133 ADHD children, relative to 288 healthy controls, had poorer functional connectivity within ventral and dorsal attention networks and poorer anti-correlation between task-positive networks and the DMN which was associated with a delay in functional maturation based on age by ADHD interaction findings (Sripada et al., 2014a). This evidence of poor deactivation of the DMN during cognitive task performance based on fMRI studies is in line with behavioural studies showing abnormally increased mind-wandering in ADHD patients as shown in self-ratings on mind-wandering scales (Biederman et al., 2017; Mowlem et al., 2016; Seli et al., 2015).

### 4.5 Similarities of fMRI-Neurofeedback effects on neural networks to stimulant effects on cognitive control and DMN networks

The effects of fMRI-Neurofeedback of rIFC on fronto-striatal and DMN regions interestingly resembles stimulant effects on neurofunctional deficit regions and networks in ADHD. Thus, a meta-analysis of fMRI studies of the most consistent single dose stimulant effects showed an increase of activation in rIFC, anterior cingulate cortex and striatal regions and a decrease in activation of dorsomedial frontal parts of the DMN (Rubia et al., 2014b). In individual studies, stimulants have also been shown to increase the deactivation of posterior DMN regions such as posterior cingulate cortex and precuneus (Cubillo et al., 2013). Longer-term stimulant administration is also associated with the upregulation of fronto-striatal regions during tasks of cognitive control (Hart et al., 2013; McCarthy et al., 2014; Norman et al., 2016). Furthermore, resting state and task-based functional connectivity studies show that stimulants most prominently enhance the strength of fronto-striatal neural networks and improve the anticorrelation between task-positive networks and the DMN (Cary et al., 2017; Querne et al., 2017; Rubia et al., 2009; Silk et al., 2017; Wong and Stevens, 2012). Similar upregulation effects on rIFC and other fronto-striatal areas and downregulation effects on posterior DMN regions and networks have also been observed with Atomoxetine (Bush et al., 2013; Cubillo et al., 2013, 2014; Lin and Gau, 2015; Schulz et al., 2012). Thus, the effects of fMRI-Neurofeedback-induced upregulation of rIFC in ADHD appear to have similar upregulation effects on fronto-cingulo-striatal cognitive control networks and similar downregulation effects on the DMN as stimulant and non-stimulant medication. The findings are promising as fMRI-Neurofeedback has the advantage of no side effects (Alegria et al., 2017; Thibault et al., 2017) and of potentially longer-lasting neuroplastic effects, as clinical improvements in the rIFC-Neurofeedback group were even more pronounced at 11 months follow-up than at post-training assessment with an effect size of almost 1 (Alegria et al., 2017), which is similar to the effect size of stimulant medication (Stevens et al., 2013).

## 5 Limitations

A strength of the study is that it is one of the few randomized controlled trials of fMRI-Neurofeedback that have been preregistered and the first in a pediatric clinical group. A limitation of the analysis is the small sample size. Also, the group difference analyses of the connectivity changes with rIFG and the correlation analyses were exploratory and hence multiple testing corrections were not applied. Future studies will have to replicate the findings in larger powered randomized controlled trials. Furthermore, we investigated functional connectivity changes that increased or decreased with neurofeedback on average across individuals. It cannot be ruled out that there were also functional connectivity changes in direct relation to symptom changes within individuals.

## 6 Conclusions

In conclusion, the findings of this study show that fMRI-Neurofeedback training of the upregulation of an isolated rIFC region that is a key cognitive control hub region and that is consistently dysfunctional in ADHD, has wider dynamic connectivity effects in the disorder. It leads to an upregulation of a fronto-striato-cingulate ***neural network*** of cognitive control and to a decrease in functional connectivity between rIFC and posterior DMN regions, which furthermore, were associated with and may be underlying the improvements in clinical ADHD symptoms. The findings are particularly relevant to ADHD as the reduced activation of the cognitive control systems and the poor deactivation of the DMN are key to the neurofunctional pathology of ADHD and are also underlying the mechanisms of action of stimulant medication (Rubia, 2018a, b; Rubia et al., 2014a). The findings hence show that fMRI-Neurofeedback of a key frontal dysfunction region in ADHD may be a promising neurotherapy to improve key ***neurofunctional network deficits*** in ADHD (Rubia, 2018a, b; Rubia et al., 2014a).

## Funding

This work, HB, AA and MW were supported by a grant from Action Medial Research (grant number: 1890) to KR. Additional support was provided by the National Institute for Health Research (NIHR) Biomedical Research Centre at South London and the Maudsley NHS Foundation Trust and King's College London and by the Medical Research Council (MRC) (MR/P012647/1) to KR which also supported MC. AA was supported by a Ph.D studentship from the Institute of Psychiatry, Psychology and Neuroscience, King's College London. The funders had no involvement in the collection, analysis and interpretation of data; in the writing of the report; or in the decision to submit the article for publication.

## Conflict of interests

Conflict of interest/financial disclosure: KR has received grant support for other studies from Lilly and Shire Pharmaceuticals, and speaker's honoraria from Medice and Shire. GJB has received honoraria for teaching from General Electric Healthcare, and acts as a consultant for IXICO. All other authors report no financial interests or potential conflicts of interest.

## References

[bib1] Alegria A.A., Wulff M., Brinson H., Barker G.J., Norman L.J., Brandeis D., Stahl D., David A.S., Taylor E., Giampietro V., Rubia K. (2017). Real-time fMRI neurofeedback in adolescents with attention deficit hyperactivity disorder. Hum. Brain Mapp..

[bib2] Alexander G.E., Crutcher M.D. (1990). Functional architecture of basal ganglia circuits: neural substrates of parallel processing. Trends Neurosci..

[bib3] Aminoff E.M., Kveraga K., Bar M. (2013). The role of the parahippocampal cortex in cognition. Trends Cognit. Sci..

[bib4] Arns M., de Ridder S., Strehl U., Breteler M., Coenen A. (2009). Efficacy of neurofeedback treatment in ADHD: the effects on inattention, impulsivity and hyperactivity: a meta-analysis. Clin. EEG Neurosci..

[bib5] Arnsten A., Rubia K. (2012). Neurobiological circuits regulating attention, movement and emotion and their disruptions in pediatic neuropsychiatric disorders. J. Am. Acad. Child Adolesc. Psychiatry.

[bib6] Beckmann C.F., DeLuca M., Devlin J.T., Smith S.M. (2005). Investigations into resting-state connectivity using independent component analysis. Philos. Trans. R. Soc. Lond. B Biol. Sci..

[bib7] Biederman J., Fitzgerald M., Uchida M., Spencer T.J., Fried R., Wicks J., Saunders A., Faraone S.V. (2017). Towards operationalising internal distractibility (Mind Wandering) in adults with ADHD. Acta Neuropsychiatr..

[bib8] Bird H.R., Shaffer D., Fisher P., Gould M.S., Staghezza B., Chen J.Y., Hoven C. (1993). The Columbia Impairment Scale (CIS)-Pilot findings on a measure of global impairment for children and adolescents. Int. J. Methods Psychiatr. Res..

[bib9] Bodurka J., Bandettini P. (2008). Real-time software for monitoring MRI scanner operation. Proc. of human brain mapping conference, Melbourne. Neuroimage.

[bib10] Brammer M.J., Bullmore E.T., Simmons A., Williams S.C., Grasby P.M., Howard R.J., Woodruff P.W., Rabe-Hesketh S. (1997). Generic brain activation mapping in functional magnetic resonance imaging: a nonparametric approach. Magn. Reson. Imag..

[bib11] Broyd S.J., Demanuele C., Debener S., Helps S.K., James C.J., Sonuga-Barke E.J.S. (2009). Default-mode brain dysfunction in mental disorders: a systematic review. Neurosci. Biobehav. Rev..

[bib12] Bullmore E., Long C., Suckling J., Fadili J., Calvert G., Zelaya F., Carpenter T.A., Brammer M. (2001). Colored noise and computational inference in neurophysiological (fMRI) time series analysis: resampling methods in time and wavelet domains. Hum. Brain Mapp..

[bib13] Bullmore E.T., Brammer M.J., Rabe-Hesketh S., Curtis V.A., Morris R.G., Williams S.C.R., Sharma T., McGuire P.K. (1999). Methods for diagnosis and treatment of stimulus-correlated motion in generic brain activation studies using fMRI. Hum. Brain Mapp..

[bib14] Bullmore E.T., Suckling J., Overmeyer S., Rabe-Hesketh S., Taylor E., Brammer M.J. (1999). Global, voxel, and cluster tests, by theory and permutation, for a difference between two groups of structural MR images of the brain. IEEE Trans. Med. Imag..

[bib15] Bush G., Holmes J., Shin L.M., Surman C., Makris N., Mick E., Seidman L.J., Biederman J. (2013). Atomoxetine increases fronto-parietal functional MRI activation in attention-deficit/hyperactivity disorder: a pilot study. Psychiatry Res. Neuroimaging..

[bib16] Cary R.P., Ray S., Grayson D.S., Painter J., Carpenter S., Maron L., Sporns O., Stevens A.A., Nigg J.T., Fair D.A. (2017). Network structure among brain systems in adult ADHD is uniquely modified by stimulant administration. Cerebr. Cortex.

[bib17] Castellanos F.X., Aoki Y. (2016). Intrinsic functional connectivity in attention-deficit/hyperactivity disorder: a science in development. Biol. Psychiatry Cogn. Neurosci. Neuroimaging.

[bib18] Christakou A., Murphy C., Chantiluke C., Cubillo A., Smith A., Giampietro V., Daly E., Ecker C., Robertson D., Murphy C., Rubia K. (2013). Disorder-specific functional abnormalities during sustained attention in youth with attention deficit hyperactivity disorder (ADHD) and with autism. Mol. Psychiatr..

[bib19] Christoff K., Irving Z.C., Fox K.C., Spreng R.N., Andrews-Hanna J.R. (2016). Mind-wandering as spontaneous thought: a dynamic framework. Nat. Rev. Neurosci..

[bib20] Cohen N.J. (2015). Navigating life. Hippocampus.

[bib21] Cole M.W., Repovs G., Anticevic A. (2014). The frontoparietal control system: a central role in mental Health. Neuroscientist.

[bib22] Cole M.W., Schneider W. (2007). The cognitive control network: integrated cortical regions with dissociable functions. Neuroimage.

[bib23] Conners C.K., Sitarenios G., Parker J.D.A., Epstein J.N. (1998). The revised Conners' Parent Rating Scale (CPRS-R): factor structure, reliability, and criterion validity. J. Abnorm. Child Psychol..

[bib24] Cortese S., Kelly C., Chabernaud C., Proal E., Di Martino A., Milham M.P., Castellanos F.X. (2012). Toward systems neuroscience of ADHD: a meta-analysis of 55 fMRI studies. Am. J. Psychiatry.

[bib25] Cox R.W. (1996). AFNI: software for analysis and visualization of functional magnetic resonance neuroimages. Comput. Biomed. Res..

[bib26] Cubillo A., Smith A., Barrat N., Giampietro V., Simmons A., Brammer M., Rubia K. (2013). Drug-specific laterality effects on frontal lobe activation of Atomoxetine and Methylphenidate in ADHD boys during working memory. Psychol. Med..

[bib27] Cubillo A., Smith A., Barrett N., Simmons A., Brammer M., V G., Rubia K. (2014). Shared and drug-specific effects of Atomoxetine and Methylphenidate on inhibitory brain dysfunction in medication-naive ADHD boys. Cerebr. Cortex.

[bib28] Cunill R., Castells X., Tobias A., Capella D. (2016). Efficacy, safety and variability in pharmacotherapy for adults with attention deficit hyperactivity disorder: a meta-analysis and meta-regression in over 9000 patients. Psychopharmacology.

[bib29] Damoiseaux J.S., Greicius M.D. (2009). Greater than the sum of its parts: a review of studies combining structural connectivity and resting-state functional connectivity. Brain Struct. Funct..

[bib30] de la Vega A., Yarkoni T., Wager T.D., Banich M.T. (2017). Large-scale meta-analysis suggests low regional modularity in lateral frontal cortex. Cerebr. Cortex.

[bib31] Dixon M.L., De La Vega A., Mills C., Andrews-Hanna J., Spreng R.N., Cole M.W., Christoff K. (2018). Heterogeneity within the frontoparietal control network and its relationship to the default and dorsal attention networks. Proc. Natl. Acad. Sci. U. S. A..

[bib32] Drechsler R., Straub M., Doehnert M., Heinrich H., Steinhausen H.C., Brandeis D. (2007). 1 Controlled evaluation of a neurofeedback training of slow cortical potentials in children with Attention Deficit/Hyperactivity Disorder (ADHD). Behav. Brain Funct..

[bib33] Dupaul D.G., Power T.J., Anastopoulos A.D., Reid R. (1998). ADHD Rating Scale-IV: Checklists, Norms, and Clinical Interpretations.

[bib34] Eklund A., Nichols T.E., Knutsson H. (2016). Cluster failure: why fMRI inferences for spatial extent have inflated false-positive rates. Proc. Natl. Acad. Sci. U. S. A..

[bib35] Emmert K., Kopel R., Sulzer J., Bruehl A.B., Berman B.D., Linden D.E.J., Horovitz S.G., Breimhorst M., Caria A., Frank S., Johnston S., Long Z., Paret C., Robineau F., Veit R., Bartsch A., Beckmann C.F., Van De Ville D., Haller S. (2016). Meta-analysis of real-time fMRI neurofeedback studies using individual participant data: how is brain regulation mediated?. Neuroimage.

[bib36] Fassbender C., Zhang H., Buzy W.M., Cortes C.R., Mizuiri D., Beckett L., Schweitzer J.B. (2009). A lack of default network suppression is linked to increased distractibility in ADHD. Brain Res..

[bib37] Fox M.D., Snyder A.Z., Vincent J.L., Corbetta M., Van Essen D.C., Raichle M.E. (2005). The human brain is intrinsically organized into dynamic, anticorrelated functional networks. Proc. Natl. Acad. Sci. U. S. A..

[bib38] Fusar-Poli P., Rubia K., Rossi G., Sartori G., Ballotin U. (2012). Dopamine transporter alterations in ADHD: pathophysiology or adaptation to psychostimulants? a meta-analysis. Am. J. Psychiatry.

[bib40] Geuter S., Koban L., Wager T.D. (2017). The cognitive neuroscience of placebo effects: concepts, predictions, and physiology. Annu. Rev. Neurosci..

[bib41] Gevensleben H., Holl B., Albrecht B., Schlamp D., Kratz O., Studer P., Rothenberger A., Moll G.H., Heinrich H. (2010). Neurofeedback training in children with ADHD: 6-month follow-up of a randomised controlled trial. Eur. Child Adolesc. Psychiatr..

[bib42] Gevensleben H., Moll G.H., Rothenberger A., Heinrich H. (2014). Neurofeedback in attention-deficit/hyperactivity disorder - different models, different ways of application. Front. Hum. Neurosci..

[bib43] Guo Y., Schmitz T.W., Mur M., Ferreira C.S., Anderson M.C. (2018). A supramodal role of the basal ganglia in memory and motor inhibition: meta-analytic evidence. Neuropsychologia.

[bib44] Hampson M., Scheinost D., Qiu M., Bhawnani J., Lacadie C.M., Leckman J.F., Constable R.T., Papademetris X. (2011). Biofeedback of real-time functional magnetic resonance imaging data from the supplementary motor area reduces functional connectivity to subcortical regions. Brain Connect..

[bib45] Harmelech T., Friedman D., Malach R. (2015). Differential magnetic resonance neurofeedback modulations across extrinsic (visual) and intrinsic (default-mode) nodes of the human cortex. J. Neurosci..

[bib46] Hart H., Radua J., Mataix D., Rubia K. (2012). Meta-analysis of fMRI studies of timing functions in ADHD. Neurosci. Biobehav. Rev..

[bib47] Hart H., Radua J., Mataix D., Rubia K. (2013). Meta-analysis of fMRI studies of inhibition and attention in ADHD: exploring task-specific, stimulant medication and age effects. JAMA Psychiatry.

[bib48] He Y., Wang J., Wang L., Chen Z.J., Yan C., Yang H., Tang H., Zhu C., Gong Q., Zang Y., Evans A.C. (2009). Uncovering intrinsic modular organization of spontaneous brain activity in humans. PloS One.

[bib49] Holtmann M., Sonuga-Barke E., Cortese S., Brandeis D. (2014). Neurofeedback for ADHD: a review of current evidence. Child Adolescent Psychiatr. Clinics North America.

[bib51] Hugdahl K., Raichle M.E., Mitra A., Specht K. (2015). On the existence of a generalized non-specific task-dependent network. Front. Hum. Neurosci..

[bib52] Hui M., Zhang H., Ge R., Yao L., Long Z. (2014). Modulation of functional network with real-time fMRI feedback training of right premotor cortex activity. Neuropsychologia.

[bib53] Kaufman J., Birmaher B., Brent D., Rao U., Ryan N.D. (1996). Schedule for Affective Disorders and Schizophrenia for School-age Children- Present and Lifetime Version (K-SADS-PL).

[bib54] Kim C., Cilles S.E., Johnson N.F., Gold B.T. (2012). Domain general and domain preferential brain regions associated with different types of task switching: a meta-analysis. Hum. Brain Mapp..

[bib55] Kim H. (2014). Involvement of the dorsal and ventral attention networks in oddball stimulus processing: a meta-analysis. Hum. Brain Mapp..

[bib56] Lawrence E.J., Su L., Barker G.J., Medford N., Dalton J., Williams S.C.R., Birbaumer N., Veit R., Ranganatha S., Bodurka J., Brammer M., Giampietro V., David A.S. (2014). Self-regulation of the anterior insula: reinforcement learning using real-time fMRI neurofeedback. Neuroimage.

[bib57] Lei D., Du M., Wu M., Chen T., Huang X., Du X., Bi F., Kemp G.J., Gong Q. (2015). Functional MRI reveals different response inhibition between adults and children with ADHD. Neuropsychology.

[bib58] Liddle E.B., Hollis C., Batty M.J., Groom M.J., Totman J.J., Liotti M., Scerif G., Liddle P.F. (2011). Task-related default mode network modulation and inhibitory control in ADHD: effects of motivation and methylphenidate. JCPP (J. Child Psychol. Psychiatry).

[bib59] Lin H.Y., Gau S.S. (2015). Atomoxetine treatment strengthens an anti-correlated relationship between functional brain networks in medication-naive adults with attention-deficit hyperactivity disorder: a randomized double-blind placebo-controlled clinical trial. Int. J. Neuropsychopharmacol..

[bib60] Little R.J.A., Rubin D.B. (2002). Statistical Analysis with Missing Data.

[bib61] Mak L.E., Minuzzi L., MacQueen G., Hall G., Kennedy S.H., Milev R. (2017). The default mode network in healthy individuals: a systematic review and meta-analysis. Brain Connect..

[bib62] Mason M.F., Norton M.I., Van Horn J.D., Wegner D.M., Grafton S.T., Macrae C.N. (2007). Wandering minds: the default network and stimulus-independent thought. Science.

[bib63] McCarthy H., Skokauskas N., Frodl T. (2014). Identifying a consistent pattern of neural function in attention deficit hyperactivity disorder: a meta-analysis. Psychol. Med..

[bib64] McDonald A.R., Muraskin J., Dam N.T.V., Froehlich C., Puccio B., Pellman J., Bauer C.C.C., Akeyson A., Breland M.M., Calhoun V.D., Carter S., Chang T.P., Gessner C., Gianonne A., Giavasis S., Glass J., Homann S., King M., Kramer M., Landis D., Lieval A., Lisinski J., Mackay-Brandt A., Miller B., Panek L., Reed H., Santiago C., Schoell E., Sinnig R., Sital M., Taverna E., Tobe R., Trautman K., Varghese B., Walden L., Wang R., Waters A.B., Wood D.C., Castellanos F.X., Leventhal B., Colcombe S.J., LaConte S., Milham M.P., Craddock R.C. (2017). The real-time fMRI neurofeedback based stratification of Default Network Regulation Neuroimaging data repository. Neuroimage.

[bib65] Molina B.S.G., Hinshaw S.P., Swanson J.M., Arnold L.E., Vitiello B., Jensen P.S., Epstein J.N., Hoza B., Hechtman L., Abikoff H.B., Elliott G.R., Greenhill L.L., Newcorn J.H., Wells K.C., Wigal T., Gibbons R.D., Hur K., Houck P.R., Grp M.T.A.C. (2009). The MTA at 8 Years: prospective follow-up of children treated for combined-type ADHD in a multisite study. J. Am. Acad. Child Adolesc. Psychiatry.

[bib66] Moussa M.N., Steen M.R., Laurienti P.J., Hayasaka S. (2012). Consistency of network modules in resting-state FMRI connectome data. PloS One.

[bib67] Mowlem F.D., Skirrow C., Reid P., Maltezos S., Nijjar S.K., Merwood A., Barker E., Cooper R., Kuntsi J., Asherson P. (2016). Validation of the mind excessively wandering scale and the relationship of mind wandering to impairment in adult ADHD. J. Atten. Disord..

[bib68] Murty V., Calabro F., Luna B. (2016). The role of experience in adolescent cognitive development: integration of executive, memory and mesolimbic systems. Neurosci. Biobehav. Rev..

[bib69] Nicholson A.A., Rabellino D., Densmore M., Frewen P.A., Paret C., Kluetsch R., Schmahl C., Theberge J., Neufeld R.W., McKinnon M.C., Reiss J., Jetly R., Lanius R.A. (2017). The neurobiology of emotion regulation in posttraumatic stress disorder: amygdala downregulation via real-time fMRI neurofeedback. Hum. Brain Mapp..

[bib70] Niendam T.A., Laird A.R., Ray K.L., Dean Y.M., Glahn D.C., Carter C.S. (2012). Meta-analytic evidence for a superordinate cognitive control network subserving diverse executive functions. Cognit. Affect Behav. Neurosci..

[bib71] Noreika V., Falter C., Rubia K. (2013). Timing deficits in patients with ADHD. Neuropsychologia.

[bib72] Norman L., Carlisi C.O., Lukito S., Hart H., Mataix-Cols D., Radua J., Rubia K. (2016). Comparative meta-analysis of functional and structural deficits in ADHD and OCD. JAMA Psychiatry.

[bib73] O'Halloran L., Cao Z., Ruddy K., Jollans L., Albaugh M.D., Aleni A., Potter A.S., Vahey N., Banaschewski T., Hohmann S., Bokde A.L.W., Bromberg U., Buchel C., Quinlan E.B., Desrivieres S., Flor H., Frouin V., Gowland P., Heinz A., Ittermann B., Nees F., Orfanos D.P., Paus T., Smolka M.N., Walter H., Schumann G., Garavan H., Kelly C., Whelan R. (2018). Neural circuitry underlying sustained attention in healthy adolescents and in ADHD symptomatology. Neuroimage.

[bib74] Oldfield R.C. (1971). The assessment and analysis of handedness: the Edinburgh inventory. Neuropsychologia.

[bib75] Papoutsi M., Weiskopf N., Langbehn D., Reilmann R., Rees G., Tabrizi S.J. (2018). Stimulating neural plasticity with real-time fMRI neurofeedback in Huntington's disease: a proof of concept study. Hum. Brain Mapp..

[bib76] Paret C., Kluetsch R., Zaehringer J., Ruf M., Demirakca T., Bohus M., Ende G., Schmahl C. (2016). Alterations of amygdala-prefrontal connectivity with real-time fMRI neurofeedback in BPD patients. Soc. Cognit. Affect Neurosci..

[bib77] Paret C., Ruf M., Gerchen M.F., Kluetsch R., Demirakca T., Jungkunz M., Bertsch K., Schmahl C., Ende G. (2016). fMRI neurofeedback of amygdala response to aversive stimuli enhances prefrontal-limbic brain connectivity. Neuroimage.

[bib78] Phillips R.C., Salo T., Carter C.S. (2015). Distinct neural correlates for attention lapses in patients with schizophrenia and healthy participants. Front. Hum. Neurosci..

[bib79] Querne L., Fall S., Le Moing A.G., Bourel-Ponchel E., Delignières A., Simonnot A., de Broca A., Gondry-Jouet C., Boucart M., Berquin P. (2017). Effects of methylphenidate on default-mode network/task-positive network synchronization in children with ADHD. J. Atten. Disod..

[bib80] Radua J., Ojeda del Pozo N., Gomez J., Guillen-Grima F., Ortuno F. (2014). Meta-analysis of functional neuroimaging studies indicates that an increase of cognitive difficulty during executive tasks engages brain regions associated with time perception. Neuropsychologia.

[bib81] Rae C.L., Hughes L.E., Weaver C., Anderson M.C., Rowe J.B. (2014). Selection and stopping in voluntary action: a meta-analysis and combined fMRI study. Neuroimage.

[bib82] Raichle M.E. (2015). The brain's default mode network. Annu. Rev. Neurosci..

[bib83] Raichle M.E., MacLeod A.M., Snyder A.Z., Powers W.J., Gusnard D.A., Shulman G.L. (2001). A default mode of brain function. Proc. Natl. Acad. Sci. U. S. A..

[bib84] Rosenberg M.D., Finn E.S., Scheinost D., Papademetris X., Shen X., Constable R.T., Chun M.M. (2016). A neuromarker of sustained attention from whole-brain functional connectivity. Nat. Neurosci..

[bib85] Rota G., Handjaras G., Sitaram R., Birbaumer N., Dogil G. (2011). Reorganization of functional and effective connectivity during real-time fMRI-BCI modulation of prosody processing. Brain Lang..

[bib86] Rota G., Sitaram R., Veit R., Erb M., Weiskopf N., Dogil G., Birbaumer N. (2009). Self-regulation of regional cortical activity using real-time fMRI: the right inferior frontal gyrus and linguistic processing. Hum. Brain Mapp..

[bib87] Rubia K. (2018). Cognitive neuroscience of attention deficit hyperactivity disorder (ADHD) and its clinical translation. Front. Hum. Neurosci..

[bib88] Rubia K., Banaschewski T., Coghill D., Zuddas A. (2018). Brain function in ADHD. Oxford Handbook for ADHD.

[bib89] Rubia K., Alegria A., Brinson H. (2014). Imaging the ADHD brain: disorder-specificity, medication effects and clinical translation. Expert Rev. Neurother..

[bib90] Rubia K., Alegria A., Cubillo A., Smith A.B., Radua J., Brammer M.J. (2014). Effects of stimulants on brain function in ADHD: a systematic review and meta-analysis. Biol. Psychiatry.

[bib91] Rubia K., Halari R., Cubillo A., Mohammad M., Taylor E. (2009). Methylphenidate normalises activation and functional connectivity deficits in attention and motivation networks in medication-naïve children with ADHD during a Rewarded Continuous Performance Task. Neuropharmacology.

[bib92] Rubia K., Smith A.B., Brammer M.J., Taylor E. (2003). Right inferior prefrontal cortex mediates response inhibition while mesial prefrontal cortex is responsible for error detection. Neuroimage.

[bib94] Ruiz S., Lee S., Soekadar S.R., Caria A., Veit R., Kircher T., Birbaumer N., Sitaram R. (2013). Acquired self-control of insula cortex modulates emotion recognition and brain network connectivity in schizophrenia. Hum. Brain Mapp..

[bib95] Rutter M., Bailey L., Lord C. (2003). Social Communication Question.

[bib96] Salavert J., Ramos-Quiroga J.A., Moreno-Alcázar A., Caseras X., Palomar G., Radua J., Bosch R., Salvador R., McKenna P.J., Casas M., Pomarol-Clotet E. (2015). Functional imaging changes in the medial prefrontal cortex in adult ADHD. J. Atten. Disord..

[bib97] Sarkheil P., Zilverstand A., Kilian-Hutten N., Schneider F., Goebel R., Mathiak K. (2015). fMRI feedback enhances emotion regulation as evidenced by a reduced amygdala response. Behav. Brain Res..

[bib98] Sato J.R., Salum G.A., Gadelha A., Picon F.A., Pan P.M., Vieira G., Zugman A., Hoexter M.Q., Anes M., Moura L.M., Del'Aquilla M.A.G., Amaro E., McGuire P., Crossley N., Lacerda A., Rohde L.A., Miguel E.C., Bressan R.A., Jackowski A.P. (2014). Age effects on the default mode and control networks in typically developing children. J. Psychiatr. Res..

[bib99] Scheinost D., Stoica T., Saksa J., Papademetris X., Constable R.T., Pittenger C., Hampson M. (2013). Orbitofrontal cortex neurofeedback produces lasting changes in contamination anxiety and resting-state connectivity. Transl. Psychiatry.

[bib100] Schulz K.P., Fan J., Bedard A.-C.V., Clerkin S.M., Ivanov I., Tang C.Y., Halperin J.M., Newcorn J.H. (2012). Common and unique therapeutic mechanisms of stimulant and nonstimulant treatments for attention-deficit/hyperactivity disorder. Arch. Gen. Psychiatr..

[bib101] Seli P., Smallwood J., Cheyne J.A., Smilek D. (2015). On the relation of mind wandering and ADHD symptomatology. Psychon. Bull. Rev..

[bib102] Sepulveda P., Sitaram R., Rana M., Montalba C., Tejos C., Ruiz S. (2016). How feedback, motor imagery, and reward influence brain self-regulation using real-time fMRI. Hum. Brain Mapp..

[bib103] Shaffer D., Gould M.S., Brasic J., Ambrosini P., Fisher P., Bird H., Aluwahlia S. (1983). A children's global assessment scale (CGAS). Arch. Gen. Psychiatr..

[bib106] Silk T.J., Malpas C., Vance A., Bellgrove M.A. (2017). The effect of single-dose methylphenidate on resting-state network functional connectivity in ADHD. Brain Imag. Behav..

[bib107] Sonuga-Barke E.J., Brandeis D., Cortese S., Daley D., Ferrin M., Holtmann M., Stevenson J., Danckaerts M., van der Oord S., Dopfner M., Dittmann R.W., Simonoff E., Zuddas A., Banaschewski T., Buitelaar J., Coghill D., Hollis C., Konofal E., Lecendreux M., Wong I.C., Sergeant J. (2013). Nonpharmacological interventions for ADHD: systematic review and meta-analyses of randomized controlled trials of dietary and psychological treatments. Am. J. Psychiatry.

[bib108] Sripada C., Kessler D., Fang Y., Welsh R.C., Kumar K.P., Angstadt M. (2014). Disrupted network architecture of the resting brain in attention-deficit/hyperactivity disorder. Hum. Brain Mapp..

[bib109] Sripada C.S., Kessler D., Angstadt M. (2014). Lag in maturation of the brain's intrinsic functional architecture in attention-deficit/hyperactivity disorder. Proc. Natl. Acad. Sci. U.S.A..

[bib110] Stevens J.R., Wilens T.E., Stern T.A. (2013). Using stimulants for attention-deficit/hyperactivity disorder: clinical approaches and challenges. Prim. Care Companion CNS Disord..

[bib111] Strehl U., Leins U., Goth G., Klinger C., Hinterberger T., Birbaumer N. (2006). Self-regulation of slow cortical potentials: a new treatment for children with attention-deficit/hyperactivity disorder. Pediatrics.

[bib112] Sulzer J., Haller S., Scharnowski F., Weiskopf N., Birbaumer N., Blefari M.L., Bruehl A.B., Cohen L.G., deCharms R.C., Gassert R., Goebel R., Herwig U., LaConte S., Linden D., Luft A., Seifritz E., Sitaram R. (2013). Real-time fMRI neurofeedback: progress and challenges. Neuroimage.

[bib113] Talairach J., Tournoux P. (1988). Co-planar Stereotaxic Atlas of the Brain.

[bib114] Thibault R.T., Lifshitz M., Birbaumer N., Raz A. (2015). Neurofeedback, self-regulation, and brain imaging: clinical science and fad in the service of mental disorders. Psychother. Psychosom..

[bib115] Thibault R.T., Lifshitz M., Raz A. (2016). The self-regulating brain and neurofeedback: experimental science and clinical promise. Cortex.

[bib116] Thibault R.T., MacPherson A., Lifshitz M., Roth R.R., Raz A. (2017). Neurofeedback with fMRI: a critical systematic review. Neuroimage.

[bib117] Thibault R.T., Raz A. (2017). The psychology of neurofeedback: clinical intervention even if applied placebo. Am. Psychol..

[bib118] Thomas R., Sanders S., Doust J., Beller E., Glasziou P. (2015). Prevalence of attention-deficit/hyperactivity disorder: a systematic review and meta-analysis. Pediatrics.

[bib119] Van Doren J., Arns M., Heinrich H., Vollebregt M.A., Strehl U., Loo S K. (2018). Sustained effects of neurofeedback in ADHD: a systematic review and meta-analysis. Eur. Child Adolesc. Psychiatr..

[bib120] van Rooij D., Hoekstra P.J., Mennes M., von Rhein D., Thissen A.J.A.M., Hestenfeld D., Zwiers M.P., Faraone S.V., Oostertaan J., Franke B., Rommelse N., Buitetaar J.K., Hartman C.A. (2015). Distinguishing adolescents with ADHD from their unaffected siblings and healthy comparison subjects by neural activation patterns during response inhibition. Am. J. Psychiatry.

[bib121] Veit R., Singh V., Sitaram R., Caria A., Rauss K., Birbaumer N. (2012). Using real-time fMRI to learn voluntary regulation of the anterior insula in the presence of threat-related stimuli. Soc. Cognit. Affect Neurosci..

[bib122] Wang G.J., Volkow N.D., Wigal T., Kollins S.H., Newcorn J.H., Telang F., Logan J., Jayne M., Wong C.T., Han H., Fowler J.S., Zhu W., Swanson J.M. (2013). Long-term stimulant treatment affects brain dopamine transporter level in patients with attention deficit hyperactive disorder. PloS One.

[bib123] Wechsler D. (1999). Wechsler Abbreviated Scale of Intelligence.

[bib124] Wehmeier P.M., Dittmann R.W., Schacht A., Helsberg K., Lehmkuhl G. (2009). Morning and evening behavior in children and adolescents treated with atomoxetine once daily for attention-deficit/hyperactivity disorder (ADHD): findings from two 24-week, open-label studies. Child Adolesc. Psychiatr. Ment. Health.

[bib125] Willcutt E.G., Doyle A.E., Nigg J.T., Faraone S.V., Pennington B.F. (2005). Validity of the executive function theory of attention-deficit/hyperactivity disorder: a meta-analytic review. Biol. Psychiatry.

[bib126] Wong C.G., Stevens M.C. (2012). The effects of stimulant medication on working memory functional connectivity in attention-deficit/hyperactivity disorder. Biol. Psychiatry.

[bib127] Yamashita A., Hayasaka S., Kawato M., Imamizu H. (2017). Connectivity neurofeedback training can differentially change functional connectivity and cognitive performance. Cerebr. Cortex.

[bib128] Yarkoni T., Poldrack R.A., Nichols T.E., Van Essen D.C., Wager T.D. (2011). Large-scale automated synthesis of human functional neuroimaging data. Nat. Methods.

[bib129] Young K.D., Siegle G.J., Misaki M., Zotev V., Phillips R., Drevets W.C., Bodurka J. (2018). Altered task-based and resting-state amygdala functional connectivity following real-time fMRI amygdala neurofeedback training in major depressive disorder. Neuroimage Clin..

[bib130] Yuan H., Young K.D., Phillips R., Zotev V., Misaki M., Bodurka J. (2014). Resting-state functional connectivity modulation and sustained changes after real-time functional magnetic resonance imaging neurofeedback training in depression. Brain Connect..

[bib131] Zhang G., Yao L., Shen J., Yang Y., Zhao X. (2015). Reorganization of functional brain networks mediates the improvement of cognitive performance following real-time neurofeedback training of working memory. Hum. Brain Mapp..

[bib132] Zhang Q., Zhang G., Yao L., Zhao X. (2015). Impact of real-time fMRI working memory feedback training on the interactions between three core brain networks. Front. Behav. Neurosci..

[bib133] Zilverstand A., Sorger B., Slaats-Willemse D., Kan C.C., Goebel R., Buitelaar J.K. (2017). fMRI neurofeedback training for increasing anterior cingulate cortex activation in adult attention deficit hyperactivity disorder. An exploratory randomized, single-blinded study. PLoS One.

[bib134] Zotev V., Phillips R., Young K.D., Drevets W.C., Bodurka J. (2013). Prefrontal control of the amygdala during real-time fMRI neurofeedback training of emotion regulation. PloS One.

