# Supplementary material for: Functional connectivity changes associated with fMRI neurofeedback of right inferior frontal cortex in adolescents with ADHD
Source: Neuroimage. 2019 Mar;188:43–58. doi: 10.1016/j.neuroimage.2018.11.055 (PMC6414400; doi:10.1016/j.neuroimage.2018.11.055)
Supplement: Multimedia component 1 [file mmc1.docx]

|  | | | **Absolute mean displacement** | | **Relative mean displacement** | |
| --- | --- | --- | --- | --- | --- | --- |
|  |  |  | r | p | r | p |
| **A. rIFC-Neurofeedback group** | | | | | | |
| **Brodmann area**  **45** | Increased connectivity | **Dorsal caudate/ACC** | -0.242 | 0.333 | -.121 | 0.632 |
|  | Decreased connectivity | **PhG** | 0.008 | 0.975 | 0.082 | 0.748 |
|  |  | **Lingual gyrus** | -0.103 | 0.684 | -0.011 | 0.966 |
|  |  | **PCC/Precuneus** | 0.081 | 0.750 | 0.077 | 0.762 |
|  |  | **Thalamus/Basal ganglia** | -0.101 | 0.689 | -0.75 | 0.768 |
| **Brodmann area**  **44** | Increased connectivity | **ACC** | -0.393 | 0.107 | -0.275 | 0.113 |
|  | Decreased connectivity | **PCC/Precuneus** | 0.116 | 0.646 | 0.270 | 0.656 |
| **B. lPHG-Neurofeedback control group** | | | | | | |
| **Brodmann area**  **36** | Decreased connectivity | **Cerebellum** | 0.113 | 0.714 | 0.158 | 0.607 |
| **Brodmann area**  **35** | Decreased connectivity | **ACC** | -0.427 | 0.146 | -0.480 | 0.097 |
| **Brodmann area**  **30** | Increased connectivity | **Paracentral gyrus** | -0.073 | 0.813 | -0.062 | 0.841 |

**Table S1. Correlations between the motion parameters and the changes in functional connectivity in the active group.**

r = Pearson correlation coefficient; p = probability value. ACC = anterior cingulate cortex; PCC = posterior cingulate cortex; PHG = parahippocampal gyrus.

| **Brain regions** | **Brodmann area** | **Peak Talairach coordinates (x;y;z)** | **Cluster size (voxels)** | **Cluster p-value** |
| --- | --- | --- | --- | --- |
| **A. Seed region: Brodmann area 36** |  |  |  |  |
| *Decreased connectivity* |  |  |  |  |
| L & R cerebellum, fusiform gyrus, parahippocampal gyrus, midbrain | 37/36/30/18/19 | -33; -41; -23 | 722 | 0.000526 |
| **B. Seed region: Brodmann area 35** |  |  |  |  |
| *Decreased connectivity* |  |  |  |  |
| R & L anterior cingulate, R middle/superior frontal gyrus | 24/32/8/9 | 18; 26; 30 | 129 | 0.007082 |
| **C. Seed region: Brodmann area 30** |  |  |  |  |
| *Increased connectivity* |  |  |  |  |
| L & R paracentral gyrus, precuneus, superior parietal lobule, paracentral gyrus, SMA, pre-, postcentral gyrus | 5/7/40/6/4 | -7; -26; 46 | 451 | 0.001076 |

**Table S2.** **Summary of changes in positive and negative functional connectivity with seed regions of the PHG**

|  |  |  | **ADHD-RS Inattention** | | **ADHD-RS Hyperactive/Impulsive** | | **CPRS-S Inattentive** | | **CPRS-R Hyperactive/Impulsive** | |
| --- | --- | --- | --- | --- | --- | --- | --- | --- | --- | --- |
|  |  |  | r | p | r | p | r | p | r | p |
| **Brodmann**  **area 36** | Decreased connectivity | **Cerebellum** | 0.025 | 0.935 | 0.062 | 0.840 | 0.046 | 0.881 | -0.139 | 0.650 |
| **Brodmann area 35** | Decreased connectivity | **ACC** | 0.080 | 0.794 | -0.039 | 0.899 | -0.181 | 0.555 | -0.354 | 0.235 |
| **Brodmann area 30** | Increased connectivity | **PCC/Precuneus** | -0.040 | 0.897 | 0.076 | 0.805 | 0.250 | 0.410 | -0.167 | 0.587 |

**Table S3. Correlations between clinical changes in the ADHD-RS and the CPRS-R and the significant functional connectivity changes in the control group.**

r = Pearson correlation coefficient; p = probability value. ACC = anterior cingulate cortex; PCC = posterior cingulate cortex.


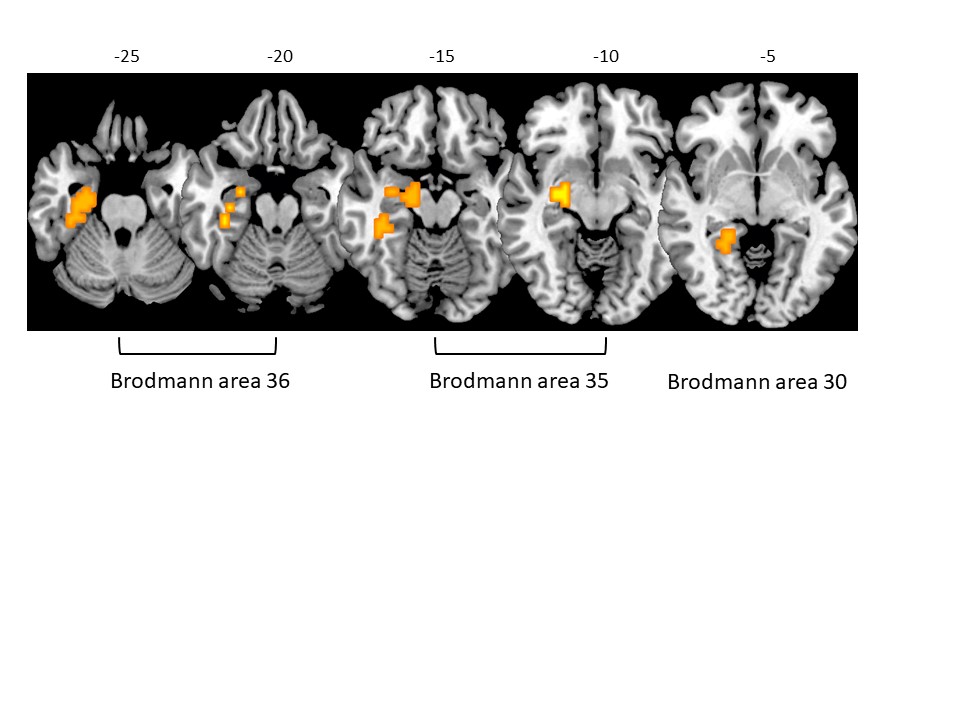


**Figure S1.** Axial slices showing progressively increased activation across 11 fMRI-neurofeedback runs in 3 regions of left parahippocampal gyrus in the control group relative to the active group, in Brodmann areas 36, 35 and 30 (adapted from Alegria et al., 2017).


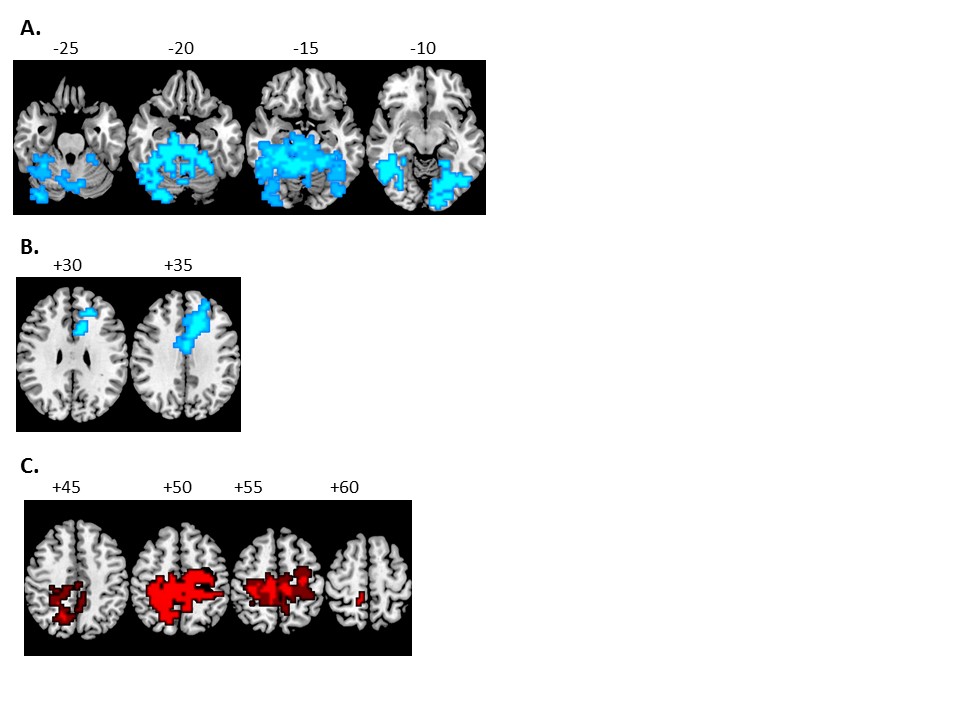


**Figure S2. Brain regions that showed increased or decreased functional connectivity with regions of the parahippocampal gyrus that were progressively increased in activation in the control group across the 11 fMRI-Neurofeedback runs. A.** Cerebellar regions that showed negative functional connectivity with Brodmann area 36. **B.** Negative functional connectivity between Brodmann area 35 and dorsal anterior cingulate. **C.** Positive functional connectivity between Brodmann area 30 and posterior cingulate and precuneus.
